# Mutations in PIH proteins MOT48, TWI1 and PF13 define common and unique steps for preassembly of each, different ciliary dynein

**DOI:** 10.1371/journal.pgen.1009126

**Published:** 2020-11-03

**Authors:** Ryosuke Yamamoto, Shiho Yanagi, Masahito Nagao, Yuya Yamasaki, Yui Tanaka, Winfield S. Sale, Toshiki Yagi, Takahide Kon

**Affiliations:** 1 Department of Biological Sciences, Graduate School of Science, Osaka University, Osaka, Japan; 2 Department of Cell Biology, School of Medicine, Emory University, Atlanta, Georgia, United States of America; 3 Faculty of Life and Environmental Sciences, Prefectural University of Hiroshima, Shobara, Hiroshima, Japan; Washington University School of Medicine, UNITED STATES

## Abstract

Ciliary dyneins are preassembled in the cytoplasm before being transported into cilia, and a family of proteins containing the PIH1 domain, PIH proteins, are involved in the assembly process. However, the functional differences and relationships between members of this family of proteins remain largely unknown. Using *Chlamydomonas reinhardtii* as a model, we isolated and characterized two novel *Chlamydomonas* PIH preassembly mutants, *mot48-2* and *twi1-1*. A new allele of *mot48* (*ida10*), *mot48-2*, shows large defects in ciliary dynein assembly in the axoneme and altered motility. A second mutant, *twi1-1*, shows comparatively smaller defects in motility and dynein assembly. A double mutant *mot48-2; twi1-1* displays greater reduction in motility and in dynein assembly compared to each single mutant. Similarly, a double mutant *twi1-1; pf13* also shows a significantly greater defect in motility and dynein assembly than either parent mutant. Thus, MOT48 (IDA10), TWI1 and PF13 may define different steps, and have partially overlapping functions, in a pathway required for ciliary dynein preassembly. Together, our data suggest the three PIH proteins function in preassembly steps that are both common and unique for different ciliary dyneins.

## Author summary

Motile cilia are hair-like organelles that protrude from many eukaryotic cells, and play vital roles in organisms including cell motility, environmental sensing and removal of infectious materials. Motile cilia are driven by gigantic motor protein complexes, called ciliary dyneins, defects in which cause abnormal ciliary motility, ultimately resulting in human diseases collectively called primary ciliary dyskinesia (PCD). Ciliary dyneins are preassembled in the cytoplasm before being transported into cilia, and preassembly requires a family of potential co-chaperones, the PIH proteins. Mutations in the PIH proteins cause defective assembly of ciliary dyneins and can result in PCD. However, despite their importance, the precise functions, and functional relationships, between the PIH proteins are unclear. In this study, using *Chlamydomonas reinhardtii*, we assessed the functional relationship between three PIH proteins with respect to dynein preassembly and motility. We found that these PIH proteins have complicated and related roles in dynein assembly, possibly with each playing common and unique roles in dynein assembly. Our results provide new information on each conserved PIH protein for dynein assembly and provide a new understanding of PCD caused by PIH mutations.

## Introduction

Motile cilia (also interchangeably referred to as flagella) are intriguing antenna-like organelles that play various important roles in eukaryotes [1, 2]. In lower eukaryotes such as *Paramecium* and *Trypanosoma*, these organelles play an indispensable role in cell motility. In higher eukaryotes including humans, cilia are essential for proper development, fertilization, and homeostasis. Defects in ciliary motility cause various symptoms including *situs inversus*, infertility, congenital heart disease and hydrocephalus in humans, collectively called as primary ciliary dyskinesia (PCD) [3, 4]. While diagnosis of PCD has attracted a good deal of attention, diagnosis can be difficult and a permanent treatment for PCD has not been established [5, 6].

The motility of cilia is driven by gigantic motor-protein complexes, referred to as ciliary dyneins that are composed of several subunits (HC: heavy chain, IC: intermediate chain, LC: light chain) and located on ciliary microtubules [1, 7–9]. Ciliary dyneins are classified into two major classes: outer dynein arm (ODA) and inner dynein arm (IDA). ODAs are particularly important for the high beat frequency of cilia, whereas IDAs are essential for creating a proper ciliary waveform [10]. Large ciliary components, including ciliary dyneins, are first assembled in the cytoplasm before being transported into and within the cilia by the intra-flagellar transport (IFT) mechanism [11–13]. This process is referred to as cytoplasmic preassembly, and many factors that are essential for the preassembly of ciliary dyneins (referred to as preassembly factors) have been reported increasing our understanding of this enigmatic process [14, 15]. Moreover, defects in the preassembly of ciliary dyneins understandably cause motility defects in cilia, resulting in PCD in humans [16–20]. In spite of its importance, the detailed mechanism of ciliary dynein preassembly in the cytoplasmic compartment, and the relationship between each preassembly factor, largely remain obscure.

In recent studies aimed at understanding of the dynein preassembly mechanism, a family of chaperone-cofactor-like proteins referred to as PIH proteins, which contain a Protein Interacting with HSP90 1 (PIH1) domain, have been shown to be tightly linked to this process [13, 17, 21] (Table 1). In vertebrates, at least four main PIH proteins (DNAAF2/KTU, PIH1D1, PIH1D2, and DNAAF6/PIH1D3) have been identified to date [13, 21, 22], and each protein has been shown to play a role in ciliary dynein preassembly [22] (Table 1), possibly residing in cytoplasmic complexes including the dynein axonemal particles (DynAPs) [23]. In *Chlamydomonas reinhardtii*, a ciliated green alga, PF13, a DNAAF2/KTU orthologue, has been shown to play an important role in the preassembly of ODAs as well as one species of IDA (IDA c)[13, 17]. Another PIH protein, MOT48 (also known as IDA10) has been shown to be necessary for the preassembly of the ODAs and a fraction of several IDA species (IDAs b, c, d, and e)[13]. In addition, a third PIH protein, TWI1 was identified in *Chlamydomonas* as an orthologue of DNAAF6/PIH1D3 [13, 21]. Among these three *Chlamydomonas* PIH proteins, knowledge of the precise function of MOT48 and TWI1 is limited, partly due to the lack of mutant alleles of the genes encoding the PIH proteins. Only the original mutant allele of *mot48*, *mot48-1* (*ida10-1*), was available for study [13]. A recent report briefly described the phenotype of a *twi1* mutant found in the CLiP library [24] as similar to wild-type [25], suggesting that there is no relationship between TWI1 and dynein preassembly. However, neither detailed study of ciliary dynein assembly nor an examination of the relationship of TWI1 to the other PIH proteins has been performed.

**Table 1:** PIH proteins involved in dynein preassembly.

Protein Name	Dynein Defects Caused by a Single PIH Mutation	Organism	Reference
**PIH1D1**	ODA, IDA “c”	*Danio rerio*	[22]
**MOT48/IDA10** ^**a**^	ODA, IDAs “b, c, d, e”, Some minor dyneins	*Chlamydomonas reinhardtii*	This study, [13, 26]
**PIH1D2**	ODA	*Danio rerio*	[22]
**DNAAF6/PIH1D3**	ODA, IDAs “f, g”	*Homo sapiens*	[18, 19, 27]
**DNAAF6/PIH1D3**	ODA and IDAs	*Mus musculus*	[21, 27]
**PIH1D3/Twister**	ODA, IDAs “c, d, g”	*Danio rerio*	[22]
**TWI1** ^**a**^	IDA “c”	*Chlamydomonas reinhardtii*	This study
**DNAAF2/KTU**	ODA and IDAs	*Homo sapiens*	[17, 27]
**KTU**	ODA and IDAs	*Oryzias latipes*	[17]
**KTU**	IDA “c”	*Danio rerio*	[22]
**PF13** ^**a**^	ODA, IDAs “b, c, g”, Minor dynein “DHC11”	*Chlamydomonas reinhardtii*	This study, [17, 26]

^**a**^ For dynein defects in a single *Chlamydomonas* PIH mutant, dynein species which showed > 30% reduction in spectral numbers compared to wild-type in this study were included.

In this study, we report the isolation/characterization of a *twi1-1* mutant and a new allele of the *mot48*, *mot48-2*. The *mot48-2* mutants swim more slowly than wild-type and have a large defect in assembly of dyneins in the ciliary axoneme. The *twi1-1* mutant has only a slightly reduced motility, as previously described [25], and has only a slight defect in dynein assembly in the axoneme. In addition, a double mutant *mot48-2; twi1-1* are more severely defective in motility and dynein assembly than either of the parent PIH mutant strains. Similarly, the double mutant *pf13; twi1-1* also shows a more severe phenotype than the parent, single mutants. These results strongly suggest that PIH proteins MOT48, TWI1 and PF13 define different steps, and have partially overlapping functions, in a pathway required for ciliary dynein preassembly.

## Results

### Isolation and characterization of *mot48-2*, a novel allele of *mot48* (*ida10*)

**Fig 1:**
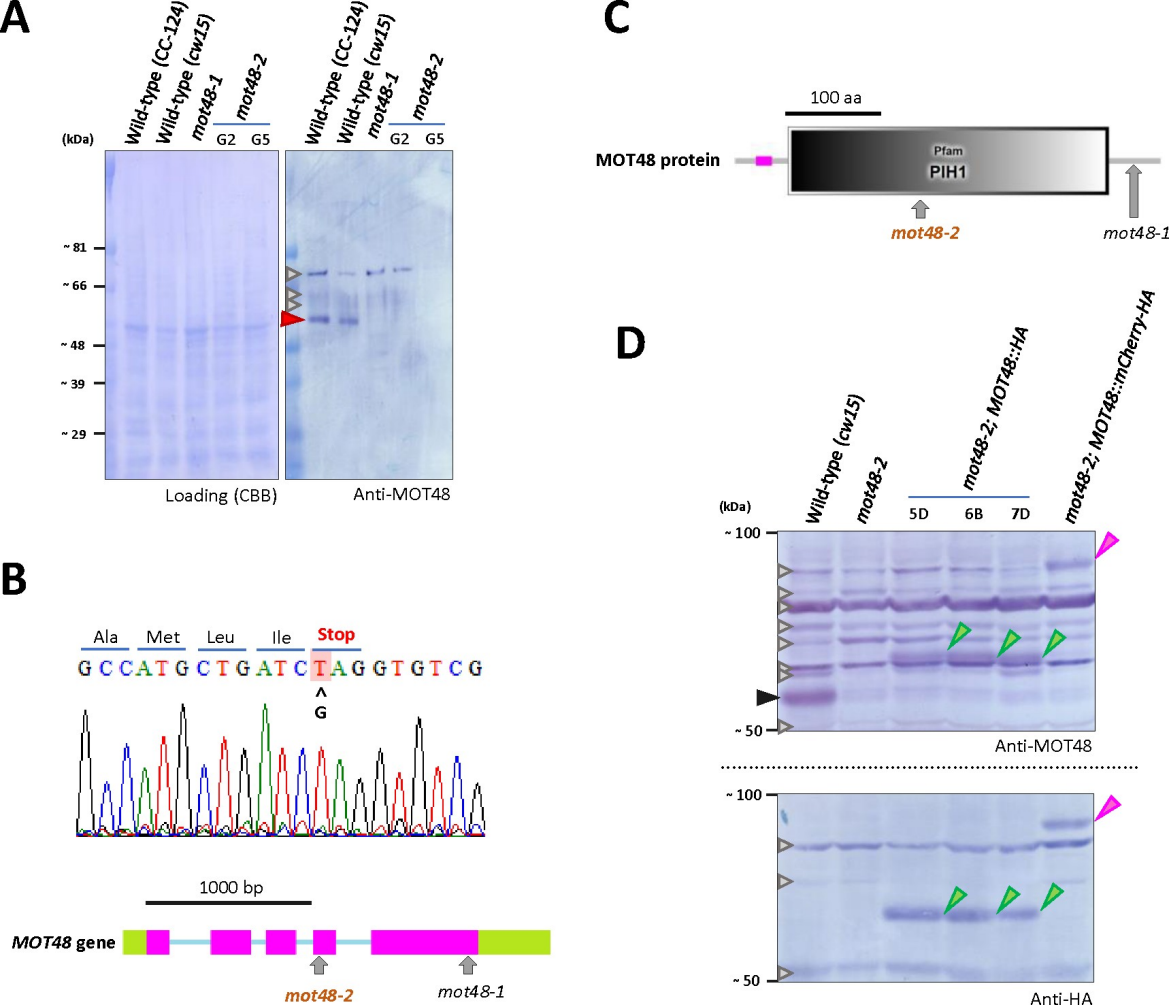
Characterization of a novel *mot48* allele, *mot48-2*. **A)** Immunoblotting analysis of whole cell samples from wild-type (CC-124, *cw15*), *mot48-1*, and two clones of *mot48-2* (G2/G5) using an anti-MOT48 antibody. The MOT48 protein band (red arrowhead) is missing in all *mot48* strains, although we cannot completely rule out the possibility that tiny amounts of MOT48 are expressed in an altered form in *mot48-1*. Gray arrowheads: non-specific bands. **B)** Sequence analysis of the *mot48-2* genomic DNA identified a point mutation (G>T) in the fourth exon of MOT48, resulting in a premature stop codon. The *MOT48* genomic structure is based on/from Phytozome (v5.5: https://phytozome.jgi.doe.gov/pz/portal.html#!info?alias = Org_Creinhardtii) and JGI (v4: https://genome.jgi.doe.gov/Chlre4/Chlre4.home.html) *Chlamydomonas* genome databases (Pink: Exon, Blue: Intron, Green: UTR). The mutation sites in *mot48-1* and *mot48-2* are indicated. **C)** The molecular structure of the MOT48 protein was predicted using a SMART analysis (http://smart.embl-heidelberg.de/). MOT48 has a PIH1 domain in the middle of its structure (gray). The pink bar represents a low-complexity region. The *mot48-1* mutant has a mutation near the C-terminus of the MOT48 molecule [13], while the new allele (*mot48-2*) has a mutation in the middle of the molecule. **D)** Immunoblot analyses of whole cell samples of wild-type (*cw15*), *mot48-2*, three independent colonies of *mot48-2; MOT48*::*HA* (5D, 6B, 7D) and one colony of *mot48-2; MOT48*::*mCherry-HA* using the anti-MOT48 and anti-HA antibodies. The MOT48 protein band (black arrowhead) present in the wild-type strain is absent in *mot48-2*. In the *mot48-2; MOT48*::*HA* strain, exogeneous MOT48 with a 3HA tag is expressed in all three independent colonies (green arrowheads). In the *mot48-2; MOT48*::*mCherry-HA*, exogeneous MOT48 with a large mCherry-3HA tag is expressed (pink arrowhead), partially rescuing the Mot48 (Ida10) phenotype. Gray arrowheads: non-specific bands.

We identified a slow swimming strain (LMJ.RY0402.055540) in the *Chlamydomonas* mutant library (CLiP) [24], with a swimming pattern that was reminiscent of the *mot48-1* mutant previously described [13]. The strain carried an additional mutation other than the original *APHVIII* insertion used to establish the library [24]. After back-crossed to wild type (CC-125) cells, we determined the MOT48 protein [13, 28] is indeed missing from mutant progeny from the cross (Fig 1A). We examined the *MOT48* sequence in the mutant progeny, and found a mutation (G>T) in the fourth exon, which results in a pre-mature stop codon. Thus, we named the new mutant allele *mot48-2* (Fig 1B) [13]. Immunoblots of whole cell samples from *mot48-1* and *mot48-2* show no evidence of MOT48 (Fig 1A) [13]. The mutation in *mot48-2* is predicted to disrupt the PIH1 domain in MOT48, deleting the potential binding motif for chaperones including HSP90 (Fig 1C) [13, 17, 29, 30]. As has been noted for *mot48-1* [13], the *mot48-2* mutants typically swim much slower (~ 85 ± 14 μm/s) than wild-type (CC-125 = ~ 136 ± 21 μm/s), although the motility varies slightly from day to day and culture to culture. A transgene expressing the wild-type MOT48 with a 3HA tag at the C-terminus (*mot48-2; MOT48*::*HA*) rescued swimming velocity (~ 131 ± 31 μm/s) and expression of the 3HA-tagged MOT48 (Fig 1D). The Mot48 phenotype was also rescued by a transgene expressing MOT48 with an added C-terminal mCherry-3HA tag (*mot48-2; MOT48*::*mCherry-HA*)(swimming velocity = ~ 120 ± 21 μm/s)(Fig 1D and S1 Fig).

**Fig 2:**
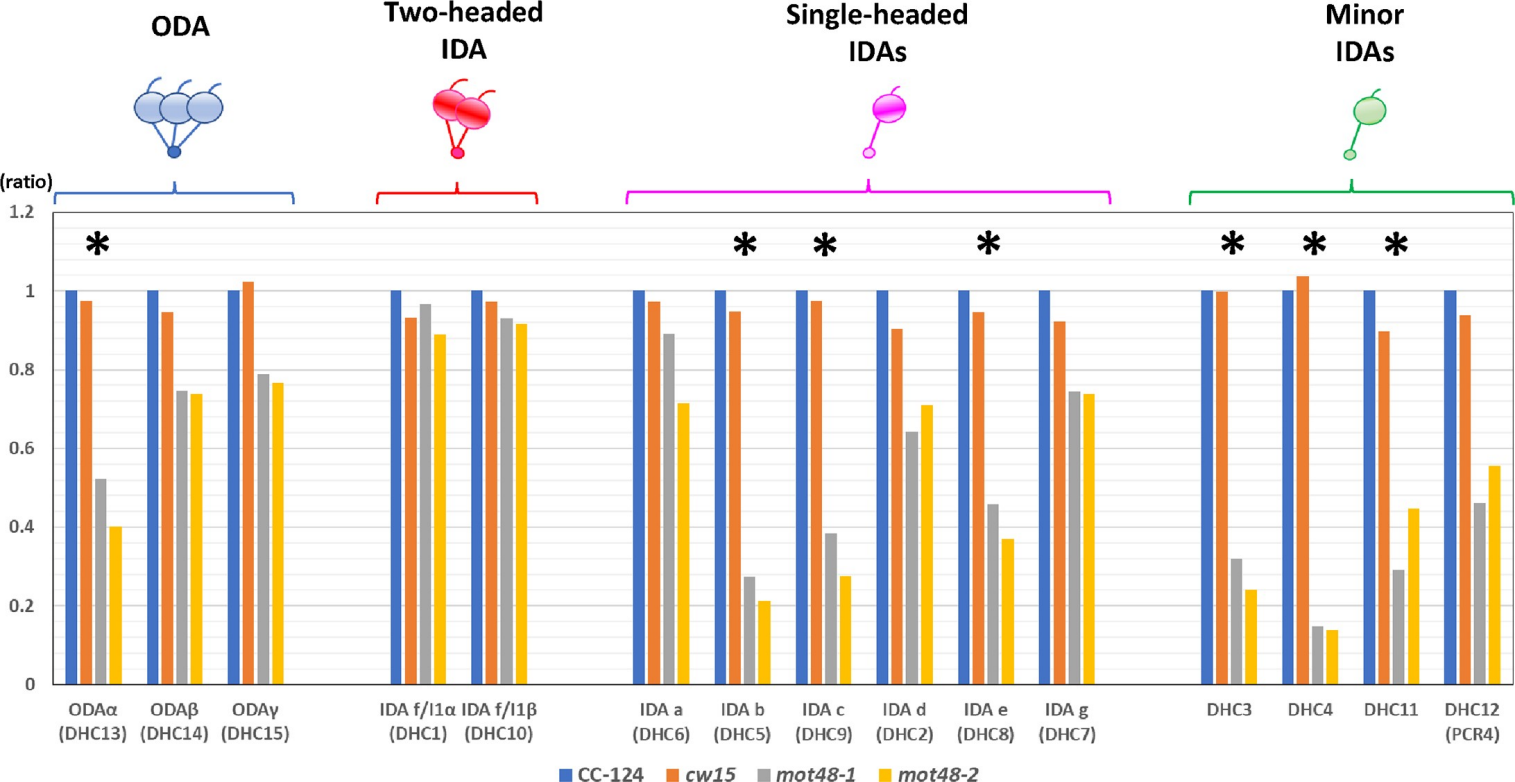
Several IDAs are deficient in the *mot48-2* ciliary axonemes. Spectral counting results (first set) of ciliary axonemal dyneins in wild-type (CC-124 and *cw15*), *mot48-1*, and *mot48-2* strains. The spectral numbers observed in the mutants were normalized using spectral numbers observed in the CC-124 wild-type strain. Asterisks indicate the ciliary axonemal dynein species for which the spectral numbers in *mot48-2* were below 50% of the levels in the CC-124 strain.

Since MOT48 has previously been reported to function in ciliary dynein preassembly and *mot48-1* cilia lacked a subset of dynein species [13], we assessed ciliary dynein assembly in the *mot48-2* mutants. To semi-quantitatively estimate the amount of each dynein species in the *mot48-2* cilia compared to wild-type (CC-124), spectral counting experiments were performed on isolated axonemes (Fig 2). Among the 15 species of ciliary dynein HCs present in the *Chlamydomonas* genome [8, 26], ODA α, IDAs b (DHC5), c (DHC9), and e (DHC8), and three minor dyneins DHC3, DHC4, and DHC11 levels are greatly reduced compared to wild-type axonemes (< 50%). In addition, ODAs β and γ, IDAs a (DHC6), d (DHC2), and g (DHC7), and one minor dynein (DHC12/PCR4 [26, 31]) show a more modest reduction in the *mot48-2* mutants (50% ~ 80% of the levels of wild type) (Relationship of dynein subunits in *Chlamydomonas* and humans is summarized in S1 Table). Immunofluorescent microscopic observation also confirmed the defects of IDA c (DHC9) and DHC11 (a minor species) in the *mot48-2* axonemes (S2 Fig). The HCs of the two-headed IDA f/I1 showed only a slight reduction in *mot48-2* mutants (Fig 2).

### Characterization of the *twi1-1* mutant, which lacks a PIH protein required for dynein preassembly

**Fig 3:**
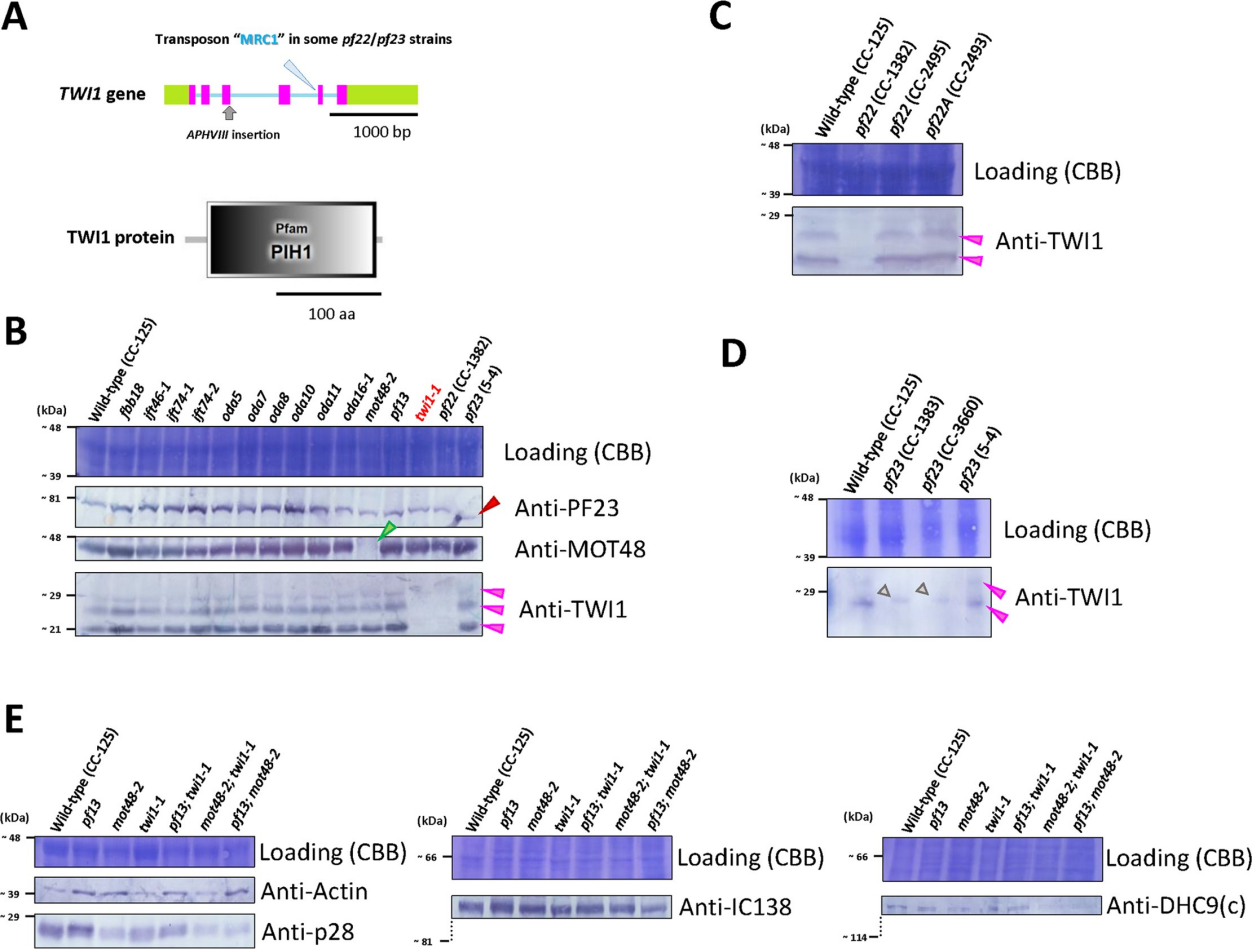
TWI1 is a dynein preassembly factor present in various ciliary mutants. **A)** Genomic structure of *Chlamydomonas TWI1* gene based on/from the Phytozome (v5.5: https://phytozome.jgi.doe.gov/pz/portal.html#!info?alias = Org_Creinhardtii) and JGI (v4: https://genome.jgi.doe.gov/Chlre4/Chlre4.home.html) genome databases (Pink: Exon, Blue: Intron, Green: UTR). The insertional mutation site in the *twi1-1* mutant is based on previous reports [24, 25]. The insertion site of the transposon, MRC1, in some of the *pf22/pf23* strains determined in this study is shown with a light blue arrowhead. The molecular structure of the TWI1 protein predicted by SMART analysis (http://smart.embl-heidelberg.de/) is also shown. TWI1 has a PIH1 domain in the middle of its structure (gray). **B)** Immunoblot of whole cell samples from various IFT-related and dynein preassembly mutants using anti-PF23, MOT48, and TWI1 antibodies. All mutants, except for *twi1-1* and *pf22* (CC-1382) show the presence of TWI1 in whole cells. As discussed in the text, TWI1 protein was visualized as consisting of two or three bands on immunoblots (pink arrowheads). The *pf23* (5–4) strain had a slightly smaller and mutated PF23 protein, as described previously (red arrowhead) [28]. The *mot48-2* mutant lacked the MOT48 protein (green arrowhead). **C)** Immunoblotting of whole cell samples from wild-type (CC-125) and three *pf22* strains (*pf22* (CC-1382), *pf22* (CC-2495), and *pf22A* (CC-2493)) using an anti-TWI1 antibody. *pf22* (CC-1382) lacked the TWI1 protein (pink arrowheads) in whole cells, because of the insertion of the *Chlamydomonas* transposon, MRC1, in the fourth intron (see **A** in this figure). **D)** Immunoblotting of whole cell samples from wild-type (CC-125) and three *pf23* strains (*pf23* (CC-1383), *pf23* (CC-3660), and *pf23* (5–4)) using the anti-TWI1 antibody. *pf23* (CC-1383) and *pf23* (CC-3660) lacked the TWI1 protein (pink arrowheads) in whole cells, because of insertion of the transposon, MRC1. *pf23* (5–4) had a normal TWI1 protein. Gray arrowheads: non-specific bands. **E)** Immunoblottings of the de-ciliated cell body samples from wild-type (CC-125) and single or double PIH mutants using the antibodies against various IDA subunits (p28/IDA4, actin/IDA5, IC138/BOP5, and DHC9/IDA c HC).

In addition to MOT48, two other PIH proteins have been identified in *Chlamydomonas* [13]. One is PF13, a protein required for ciliary dynein assembly based on characterization of the dynein-deficient mutant *pf13* [32]. Defects of its orthologue in mammals (DNAAF2/KTU) have been reported to cause the ciliopathy [17] (Table 1). The other is the TWI1 protein (Fig 3A) (predicted molecular weight = ~ 20590), and defects in the TWI1 orthologue (DNAAF6/PIH1D3) also cause the ciliopathy [18, 19, 21] (Table 1). In addition, the expression of TWI1 is highly induced upon deciliation [33]. A *twi1* strain (LMJ.RY0402.076787) was recently identified in the CliP library [24], and reported that the swimming phenotype of this LMJ.RY0402.076787 strain is similar to wild-type [25]. Thus, we back-crossed the LMJ.RY0402.076787 strain to wild-type (CC-125) and the *twi1* progeny (*twi1-1*) phenotype was characterized. Compared to wild-type *Chlamydomonas* (CC-125)(~ 136 ± 21 μm/s), the *twi1-1* swims at a slightly reduced rate (~ 103 ± 14 μm/s) after the 3-day liquid culture, suggesting *twi1-1* had subtle defects in assembly of ciliary dyneins (S1 Fig). Like other mutants defective in genes encoding PIH proteins, the swimming phenotypes vary slightly from day to day.

An antibody against TWI1 was generated, and we noticed that TWI1 shows two or three bands on immunoblots (Fig 3B-3D). Since these TWI1 bands could be observed even in freshly-boiled *Chlamydomonas* whole cell SDS-PAGE samples, we presume that these band shifts represent modified forms of TWI1, rather than protein degradation. We assessed if the TWI1 band patterns were altered following treatment with calf intestinal phosphatase, and found that no changes occurred, suggesting these shifts did not arise as a result of TWI1 phosphorylation. Also, we could not identify alternative-splicing variants of *TWI1* cDNA. One possibility is that the TWI1 protein is structurally stable, and that boiling/SDS-treatment is not sufficient to completely denature the protein, as has been observed for another preassembly factor CCDC103 [34].

**Fig 4:**
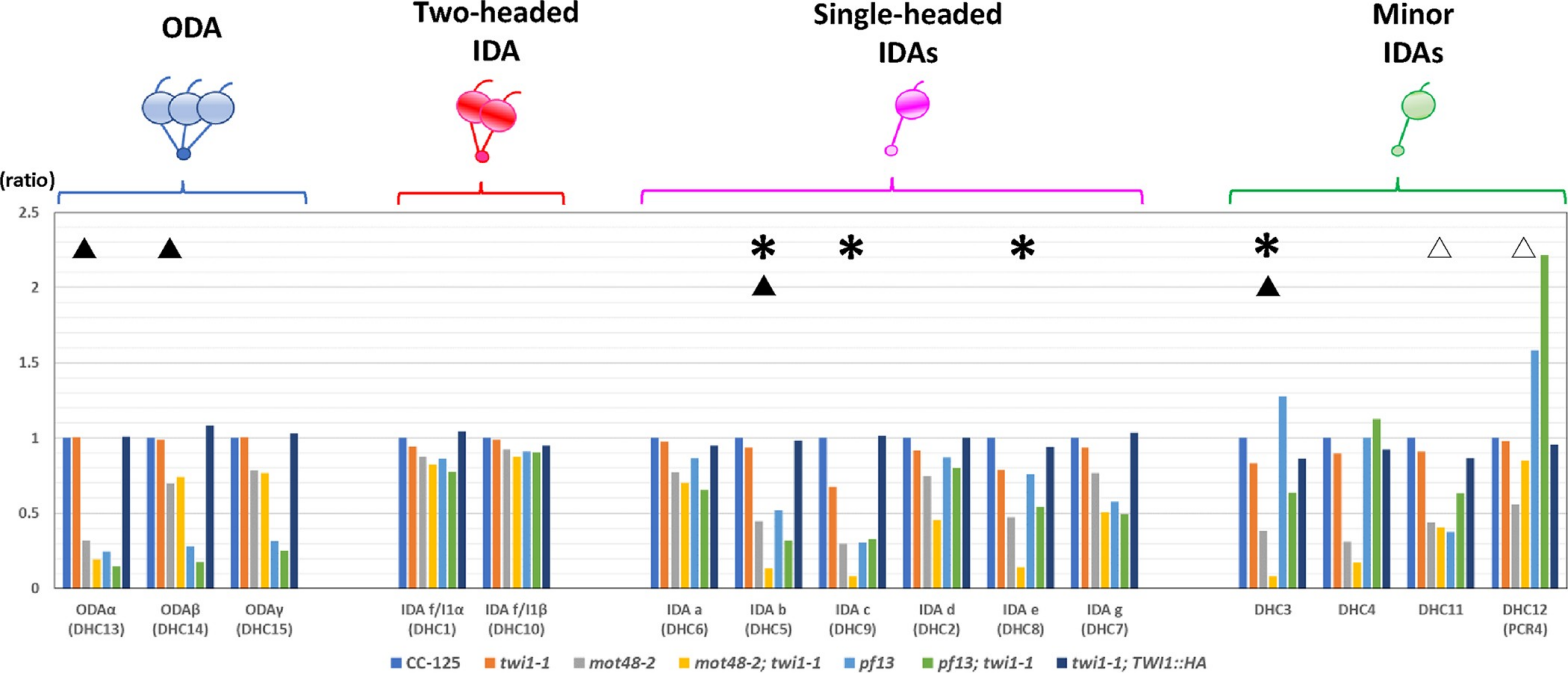
Dynein defects are profound in double PIH preassembly mutants. Spectral counting results (second and third sets combined) of ciliary axonemal dyneins from wild-type (CC-125), *twi1-1*, *mot48-2*, *mot48-2; twi1-1*, *pf13*, *pf13; twi1-1* and *twi1-1; TWI1*::*HA*. The spectral numbers observed in the mutants were normalized using the spectral numbers from the CC-125 strain. Asterisks indicate the ciliary dynein species for which the spectral numbers in the *mot48-2; twi1-1* strain showed more than a 50% reduction compared to the *mot48-2* strain. The black triangles indicate the ciliary dynein species for which the spectral numbers in the *pf13; twi1-1* strain showed a more than 30% reduction compared to the *pf13* strain. DHC11 and DHC12 showed ~ 50% increase in *pf13; twi1-1* compared to *pf13* (white triangles).

To test if the IFT-related or preassembly-related mutations affect the stability of the TWI1 proteins, we performed the immunoblots using the TWI1 antibody on the whole cell samples from various IFT-related and preassembly-related mutants. Immunoblots reveal the TWI1 protein is present in most IFT-related and preassembly-related mutants (e.g. *ift46-1*, *ift74-1*, and *oda5*)(Fig 3B)(See S1 Table summarizing *Chlamydomonas* and human proteins). Surprisingly, *pf22* (CC-1382) and *pf23* (CC-1383 and CC-3660) strains completely lack TWI1 (Fig 3B–3D). In contrast, immunoblots of whole cell samples from *pf22* (CC-2495), *pf22A* (CC-2493), and *pf23* (5–4) strains have normal levels of TWI1 (Fig 3C and 3D). The result strongly suggested TWI1 loss in *pf22* (CC-1382) and *pf23* (CC-1383 and CC-3660) strains occurs because of an additional mutation. *TWI1* sequence in these mutant strains revealed the transposon MRC1 (~ 1,600 bp) [35] inserted in the fourth intron of the *TWI1* gene (Fig 3A). Since these strains were first isolated in Dr. David Luck’s laboratory in the 1970’s [32], we suspect that some of the parent strains used for mutagenesis in Luck laboratory had this transposon insertion in *TWI1* gene, and that these strains (*pf22* (CC-1382) and *pf23* (CC-1383 and CC-3660)) are actually double mutants lacking their respective proteins (*Chlamydomonas* PF22 or PF23) and TWI1. Other *pf* strains from Luck laboratory might also carry this *twi1* (*twi1-2*) background. We carried out spectral counting experiments (Fig 4) of the ciliary axonemal dyneins in the *twi1-1* mutant, and found that only the levels of IDAs c (DHC9) and e (DHC8) were modestly reduced compared to wild-type (CC-125). This observation is consistent with the mild motility phenotype of *twi1-1*.

### *Chlamydomonas* PIH proteins MOT48, TWI1, and PF13 have overlapping and unique roles in assembly of different ciliary dyneins

We took advantage of *Chlamydomonas* genetics by isolating double PIH mutants (*mot48-2; pf13*, *mot48-2; twi1-1* and *pf13; twi1-1*) from crosses between single PIH mutants (*pf13*, *mot48-2*, and *twi1-1*). The predictions included that if deleted proteins function together in the same path and a phenotype of one mutant is similar or the same as the other, then the double mutant phenotype would nearly match the phenotype of the single mutants. Also, if deleted proteins function together in the same path but a phenotype of one mutant is more severe to the other, then the double mutant phenotype would match the phenotype of the more deleterious single mutant. Alternatively, if the deleted proteins operate in different pathways, or have some overlapping function but do not function together in the same path, then the double mutants would have a more severe phenotype than the single mutants.

The motility phenotype of the *mot48-2; twi1-1* double mutant is worse than the *mot48-2* single mutant (swimming velocity: *mot48-2* = ~ 85 ± 14 μm/s; *mot48-2; twi1-1* = ~ 49 ± 13 μm/s). Furthermore, about half of the double mutant cells have completely non-motile cilia while the other half of the cells display a slow swimming phenotype (S1 Fig). In addition, the percentage of ciliated cells of the *pf13; twi1-1* double mutant (~ 16%) is much lower than the *pf13* (~ 58%) single mutant. These observations strongly suggest that TWI1 protein is involved in ciliary dynein preassembly.

To further test the idea that dynein assembly is more defective in the double mutants, we performed spectral counting experiments on dyneins in isolated axonemes from the double and single PIH preassembly mutants to compare the amount of ciliary dyneins assembled (Fig 4). The *mot48-2; pf13* double mutant grew extremely short cilia or was missing cilia. This severe phenotype hindered comparison of the amount of ciliary dynein assembled in this strain. This short cilia phenotype was also previously observed in the *mot48-1; pf13* mutant [13]. Predictably, this short-cilia phenomenon was a consequence of pre-assembly failure of sufficient number of ciliary dyneins required for ciliary elongation.

The peptide numbers for a subset of ciliary dyneins in the *mot48-2; twi1-1* double mutant are greatly reduced compared to *mot48-2* (Fig 4). In particular, the IDAs b (DHC5), c (DHC9), and e (DHC8), and one minor dynein, DHC3 are greatly reduced in the double mutant compared to *mot48-2* alone. This result indicates that both MOT48 and TWI1 function in the preassembly of these dynein species, but in possibly different steps. Alternatively, MOT48 and TWI1 have overlapping functions in the same step of preassembly, and loss of the two PIH proteins cause severe defects for some ciliary dyneins. Peptide numbers of ODAs α and β, and IDA b (DHC5) in the *pf13; twi1-1* double mutant are modestly reduced compared to the *pf13* mutant, indicating that PF13 and TWI1 both function in the preassembly of these dyneins possibly in different steps, and/or have some overlapping function in the same step (see Discussion).

It is intriguing that peptide numbers of some minor dyneins (DHC12 in the *mot48-2; twi1-1* double mutant, and DHC11 and DHC12 in the *pf13; twi1-1* double mutant) are much greater in the double PIH mutants than in the single PIH mutants (*pf13*, *mot48-2*, and *twi1-1*)(Fig 4). This result suggests that the preassembly of these minor dynein species is not affected by the double PIH mutations and that these minor dyneins partially replace the major dynein species that are affected in these double mutants. While some major IDAs are predicted to be replaced by minor dynein species at the proximal end of the cilia [36, 37], replacement of major dynein species by minor dynein species, especially DHC12 in the double PIH mutants, must be confirmed by future biochemical studies.

In addition, to check the stability of various dynein subunits in the cytoplasm, we performed immunoblots of de-ciliated cell-body samples. Immunoblots of dynein subunits (S1 Table) on the de-ciliated cell-body samples of the single or double PIH mutants revealed that one IDA subunit, p28/IDA4 was apparently reduced in the cell bodies from PIH mutants with *mot48* background (Fig 3E). In contrast, another IDA subunit, actin/IDA5 appeared to accumulate in the cell bodies from PIH mutants with the *pf13* and/or *mot48* background (Fig 3E). The reduction in p28/IDA4 and increase in actin/IDA5 were also previously observed in *mot48-1* [13]. In addition, IDA c HC (DHC9) is reduced in the PIH mutants with the *mot48* background (Fig 3E).

**Fig 5:**
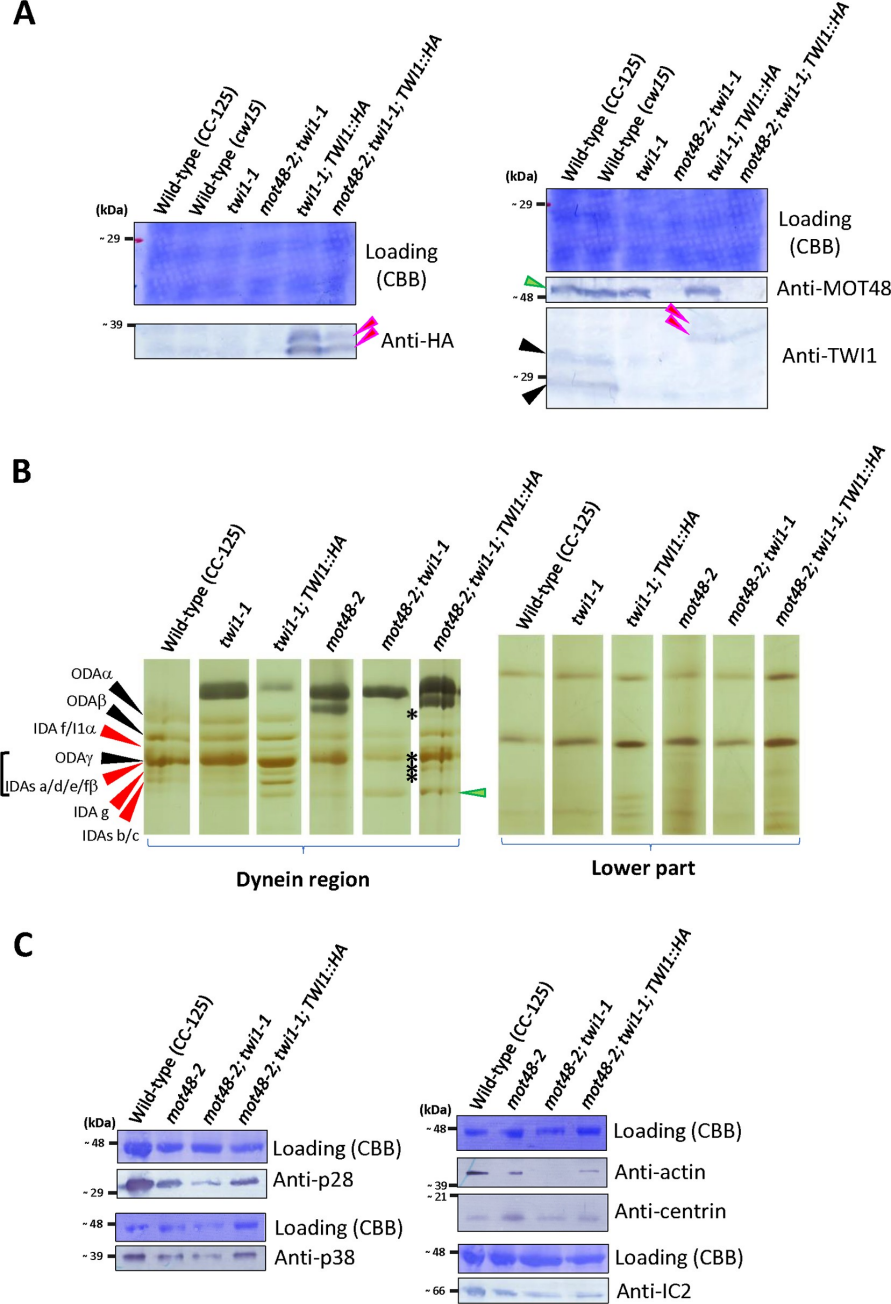
Exogeneous TWI1 protein can rescue the Twi1 phenotype. **A)** Immunoblot of whole cell samples from wild-type (CC-125 and *cw15*), *twi1-1*, *mot48-2; twi1-1*, *twi1-1; TWI1*::*HA* and *mot48-2; twi1-1; TWI1*::*HA* strains using anti-HA (left)/MOT48 and TWI1 (right) antibodies. The black arrowheads indicate the wild-type endogenous TWI1 protein. Red arrowheads indicate the exogeneous TWI1 protein with the 3HA tag. The cDNA-driven exogeneous TWI1::3HA proteins show two bands in these blots (red arrowheads). A green arrowhead indicates the MOT48 protein. **B)** Urea-PAGE of axonemes from wild-type (CC-125), *twi1-1*, *twi1-1; TWI1*::*HA*, *mot48-2*, *mot48-2; twi1-1*, and *mot48-2; twi1-1; TWI1*::*HA* strains. For presentation, gel regions of ciliary dyneins and lower parts are shown. The relative positions of ciliary dyneins were adjusted between all strains for comparison. The black arrowheads indicate the HCs of ODA. The red arrowheads indicate the HCs of IDAs. HCs of ODAγ and IDAs a, d, e and fβ form a large band in the urea gel. A green arrowhead indicates HC degradation products. In the *mot48-2; twi1-1* strain, the ODAα and IDA bands were missing (asterisks), but these dyneins were recovered in the *mot48-2; twi1-1; TWI1*::*HA* strain. The correspondence between bands in the Urea-PAGE gel and DHCs was based on [65–67]. **C)** Immunoblots of axonemal samples from wild-type (CC-125), *mot48-2*, *mot48-2; twi1-1* and *mot48-2; twi1-1; TWI1*::*HA* strains using dynein-subunit antibodies (anti-p28/IDA4, p38, actin/IDA5, centrin/VFL2, and IC2/IC69/ODA6; S1 Table).

As a further test, we performed rescue experiments to see if recovery of the TWI1 protein in the *twi1-1* and *mot48-2; twi1-1* would rescue the observed phenotypes. Exogenously expressed TWI1::3HA proteins in *twi1-1; TWI1*::*HA* and *mot48-2; twi1-1; TWI1*::*HA* (S2 Table) successfully rescue both the swimming defects (swimming velocity: *twi1-1* = ~ 103 ± 14 μm/s; *twi1-1; TWI1*::*HA* = ~ 121 ± 21 μm/s; *mot48-2; twi1-1* = ~ 49 ± 13 μm/s; *mot48-2; twi1-1; TWI1*::*HA* = ~ 71 ± 11 μm/s) and ciliary dynein assembly (Figs 4 and 5A–5C and S1 Fig). Notably, the expressed exogeneous TWI1::3HA proteins by the cDNA rescue show several forms in the immunoblots (Fig 5A), suggesting these variants most likely derive from structural differences or some modification rather than alternative splicing or protein degradation. These results indicate that the observed phenotypes in *twi1-1* and *mot48-2; twi1-1* were indeed derived from loss of the TWI1 protein.

### TWI1 may work together with other preassembly factors

In addition to the PIH mutants, we also performed spectral counting of dyneins in isolated axonemes from *pf23* (5–4) and *pf22A* (CC-2493)(which contain a wild-type *TWI1* gene) and compared to *pf23* (CC-1383) and *pf22* (CC-1382)(which contain a mutation in the *TWI1* gene, described above). The dynein defects in *pf23* (CC-1383) are more profound than *pf23* (5–4)(S3A Fig). Particularly, the defects in IDA d (DHC2) and IDA g (DHC7) are larger in *pf23* (CC-1383) than in *pf23* (5–4) [28]. On the other hand, the dynein defects in *pf22A* (CC-2493) and *pf22* (CC-1382) are relatively similar to each other (S3A Fig). In addition to ODAs and IDAs b (DHC5) and c (DHC9) as previously described [16, 32], these *pf22* mutants have large defects (< 50% of wild-type) in IDAs a (DHC6) and e (DHC8) and minor dyneins DHC3 and DHC4. Also, the axonemal amount of one minor dynein DHC12 is increased in the *pf22* mutants (S3A and S3B Fig). Given that DNAAF4/DYX1C1 (PF23 orthologue)(S1 Table) and DNAAF6/PIH1D3 (TWI1 orthologue)(Table 1) in mammals are predicted to form a complex and work together in dynein preassembly [18], the large dynein defects observed in *pf23* (CC-1383; with *twi1-2* background) may indicate that *Chlamydomonas* TWI1 is needed for efficient function of the PF23 protein in dynein preassembly. Additionally, the swimming phenotype of the *pf23* (CC-1383) strain, rescued with the wild-type *PF23* gene, which also harbored the *twi1-2* mutation, was indistinguishable from wild-type [28], consistent with the subtle swimming defect observed in the *twi1-1* mutant.

## Discussion

**Fig 6:**
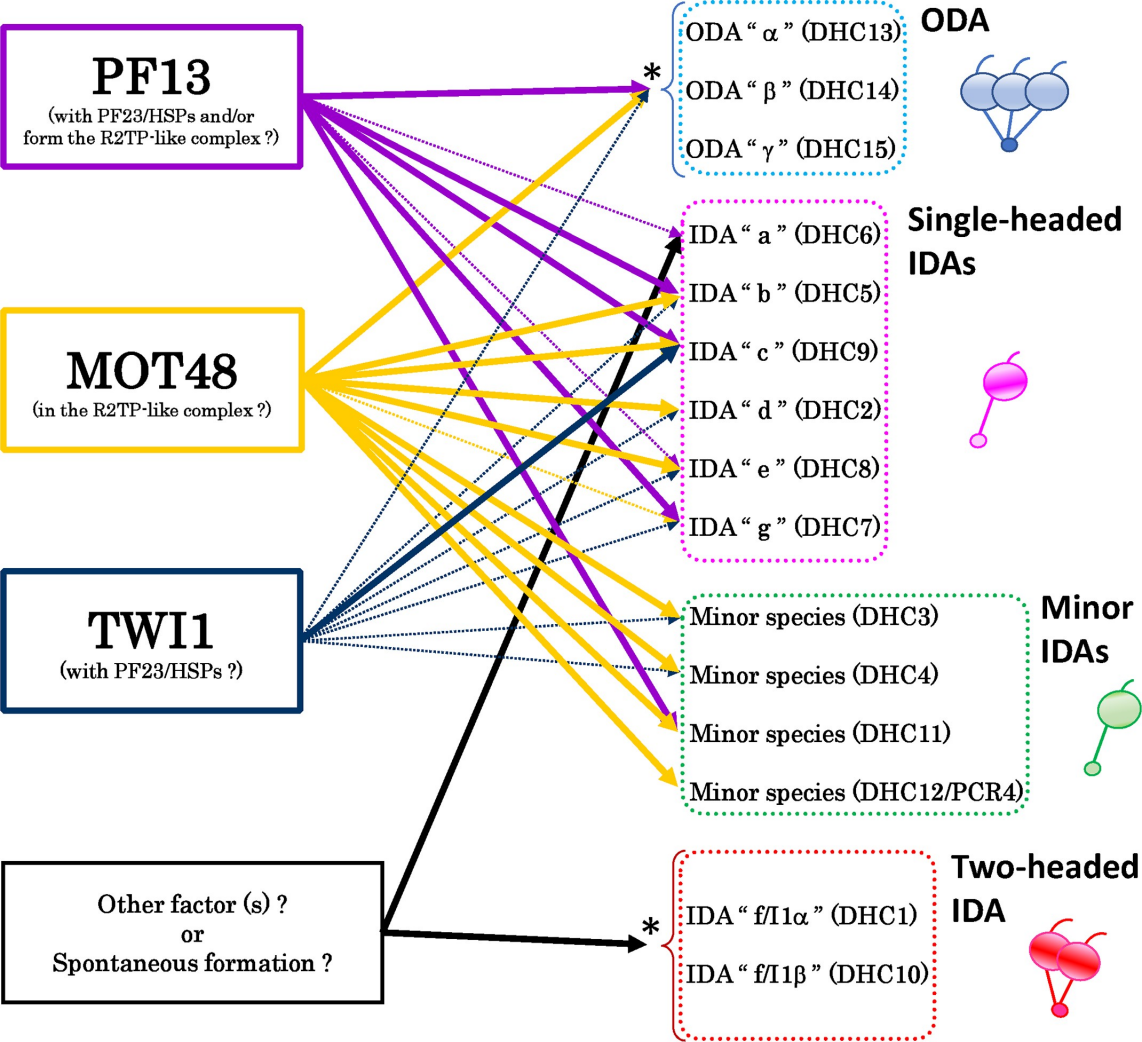
Updated model of the PIH-protein pathways in the dynein preassembly. A proposed model of the functional pathways of PIH preassembly proteins in *Chlamydomonas* adapted from [13]. The bold lines indicate the primary pathways, showing the dynein species that were reduced more than 30% in spectral numbers in each PIH mutant compared to the wild-type. The dotted thin lines indicate relatively secondary pathways, showing the dynein species that were reduced more than 30% in spectral numbers in the double PIH-mutants compared to the single PIH-mutants. The pathways are more complicated than previously thought [13]. Since the three-headed ODA of *Chlamydomonas* cannot be assembled in axonemes in the absence of ODA β or γ HC [68, 69], in this study we could not assess the direct contribution of each PIH protein in the cytoplasmic preassembly of each ODA HC since the amount of ODA α in cilia could be reduced simply in the absence of ODA β or γ HC. Thus, we categorized ODA HCs into one group in this model (asterisk). This is also the case for the two-headed IDA f/I1, in which this species cannot be assembled without each HC (f/I1α or f/I1β)[70, 71], thus we also categorized IDA f/I1 HCs into one group (asterisk). In this study, we could not find any primary pathway of the PIH proteins for the preassembly of IDAs a and f/I1. These dynein species may be preassembled by factors other than PIH proteins (e.g. PF22 or PF23, see S3 Fig), or can assemble spontaneously in cytoplasm to some extent without the help of preassembly factors.

In this report, we characterized two novel PIH preassembly mutants in *Chlamydomonas reinhardtii*, *mot48-2* and *twi1-1*. Although recent studies reveal a conserved role(s) of PIH proteins in ciliary dynein preassembly [13, 17–19, 21, 22], the specificity of their molecular function(s) and interaction(s) is not fully understood. Our study of assembly of specific dyneins in the axoneme, in the single and double PIH mutants, revealed partially overlapping and specific roles for three *Chlamydomonas* PIH proteins, MOT48, TWI1, and PF13. Accordingly, we have updated our previous model [13] of the preassembly pathway involving PIH proteins and ciliary dynein species (Fig 6). The preassembly pathway of the PIH proteins is more complicated than previously predicted [13], with assembly of each ciliary dynein requiring a specific complement of PIH proteins (Fig 6). We observed assembly defects in some dynein species are more severe in the double preassembly mutants (e.g. *mot48-2; twi1-1*, *pf13; twi1-1* and *pf23; twi1-2* (CC-1383)) than a single mutant (e.g. *mot48-2*, *pf13* and *pf23*). In addition, dynein f/I1, and possibly IDA a, do not require PIH proteins for assembly (Fig 6 and see [22]). We discuss possibilities in context to the severe phenotypes in the double PIH mutants.

Preassembly of ciliary dyneins likely requires a series of ordered steps each requiring a PIH preassembly protein. For example, full assembly of dynein species IDAs b and c in the axoneme require the activity of at least two PIH proteins including PF13, MOT48 and possibly TWI1 (Figs 4 and 6). Sequential steps in the dynein preassembly have been predicted in previous studies [38–40]. PIH proteins and other preassembly factors may be organized in complexes/organelles such as the DynAP [23] or organized in a series of individual complexes operating in ordered steps (Table 2). Thus, whether in a single complex or in a series of complexes, each PIH protein may operate in a different step in preassembly, and loss of PIH proteins would attenuate the whole preassembly process.

**Table 2:** Interacting partners of PIH proteins predicted/identified in previous studies.

Protein Name	Potential Interacting Partner^a^	Reference
**PIH1D1/MOT48/IDA10**	DNAAF4/DYX1C1	[25]
	HSP90	[18, 40, 41]
	RPAP3	[18, 25, 40–43]
	RuvBL1/Pontin	[18, 25, 40–43]
	RuvBL2/Reptin	[18, 25, 40–43]
	WDR92/Monad	[25, 40, 43]
**PIH1D2**	HSP70	[27]
	HSP90	[27]
	SPAG1	[42, 43]
	RuvBL1/Pontin	[42, 43]
	RuvBL2/Reptin	[42, 43]
**DNAAF6/PIH1D3/Twister/TWI1**	DNAAF4/DYX1C1	[18, 19, 40, 42, 43]
	DNAAF2/KTU/PF13	[19]
	HSP70	[21]
	HSP90	[18, 21]
**DNAAF2/KTU/PF13**	DNAAF4/DYX1C1	[18, 40, 42, 43]
	HSP70	[17]
	HSP90	[18]
	RuvBL1/Pontin	[18, 43]
	RuvBL2/Reptin	[18, 43]
	SPAG1	[42, 43]

^**a**^ Potential interacting partners of PIH proteins predicted/identified in various organisms are summarized in one table.

The apparent functional overlap between PIH proteins required for preassembly of certain dyneins, such as IDA c, suggests that the PIH proteins can work with partially overlapping function. Thus, loss of two PIH proteins would cause a more severe phenotype than a single mutation. Consistent with this idea, missing PIH proteins appear to be partially compensated by the other PIH proteins to some extent. For example, the apparent subtle motility defect in *twi1-1* likely derives from a compensatory function of MOT48 and PF13 in the cytoplasm of *twi1-1* cells (see IDA c in Fig 4). This hypothesis is also consistent with our observation that the motility of the *mot48-1*/*mot48-2* mutant improves as the cells in liquid culture grow old, implying that in the *mot48* cells, PF13 and TWI1 eventually compensate and help to preassemble ciliary dyneins that are usually dependent on MOT48. This idea could explain the occasional ODA assembly in *pf13* axonemes observed by Huang *et al*., [32]. Predictably, MOT48 and/or TWI1 partially compensate, with time, for PF13 in the *pf13* mutant.

As mentioned above, preassembly factors including PIH proteins, PF22 and PF23 may form in the molecular complex/organelle DynAPs [23]. Based on our data, loss of one subunit protein from this complex may have a modest effect on the activity of the whole complex, but loss of two or more specific subunits largely blocks the activity of the complex for dynein pre-assembly. Interestingly, in contrast to IDAs b and c, assembly of the minor dyneins, particularly DHC12, only seems to require MOT48. Although we focused on the assembly of dynein HCs in the axoneme, we also observed either an increase or decrease in specific LCs in the cytoplasmic compartment. For example, DHC9 (IDA c HC) and p28/IDA4, a LC of several single-headed IDAs, are reduced in the cytoplasm of PIH mutants with the *mot48* background (Fig 3E, see also [13]). In addition, actin/IDA5, another LC of single-headed dyneins, accumulates in the cytoplasm of PIH mutants with the *pf13* and/or *mot48-2* background (Fig 3E). Thus, stability of dynein subunits in the cytoplasm may offer another approach toward understanding PIH protein function. One model is that the PIH proteins play roles in the folding and stability of dynein HCs, and also in the following LC assembly to HC [17–19, 21, 40]. Further biochemical analyses, in combination with the *in vitro* reconstitution, are required to define the details of PIH protein function.

Although *Chlamydomonas* MOT48 was first identified as a protein that is conserved in organisms with motile cilia [44], an exact orthologous *MOT48* gene in vertebrates remains unclear. A recent study showed that among the four PIH proteins found in vertebrates, PIH1D1, PIH1D2, DNAAF6/PIH1D3 (TWI1 orthologue), and DNAAF2/KTU (PF13 orthologue), MOT48 groups near DNAAF2/KTU and PIH1D1 in a phylogenetic tree [22]. We also generated a phylogenetic tree using the full-length sequences of PIH proteins (S4 Fig), and also found that MOT48 fell into a group with PIH1D1 proteins. A BLAST search against the NCBI database (https://blast.ncbi.nlm.nih.gov/Blast.cgi) also revealed that among the four PIH proteins in vertebrates, PIH1D1 showed the highest similarity to MOT48, although the E-values were relatively low (< 7E-15). In addition, recently MOT48 has been reported to interact with RPAP3 and RuvBL1 [25], components of the known R2TP chaperone complex [41] to form a potential R2TP-like complex in *Chlamydomonas* cytoplasm (Table 2). Thus, MOT48 may have a function as a co-factor in the *Chlamydomonas* R2TP-like complex, which is similar to the PIH1D1 function in higher eukaryotes [41].

The TWI1 orthologue, DNAAF6/PIH1D3, has also been postulated to interact with DNAAF4/DYX1C1 [18], orthologous to the PF23 protein in *Chlamydomonas* [28]. Thus, TWI1 may function as part of a large chaperone complex [18, 19, 23](Table 2). Using the 3HA-tagged rescued strains (*mot48-2; MOT48*::*HA* and *twi1-1; TWI1*::*HA*) for identification of interacting partners, we failed to identify chaperone related proteins. This negative result could indicate weak and/or transient interactions of the PIH proteins with chaperones and/or interacting partners of MOT48 and TWI1. Further structural and biochemical studies of PIH interacting proteins, including specific dynein HC/IC/LCs, are required for understanding assembly complexes, steps and specificity of each PIH protein required for ciliary dynein assembly.

## Summary

The mechanisms for PIH proteins in assembly of ciliary dyneins are more complicated than previously thought. *Chlamydomonas* uses the three PIH proteins, MOT48, TWI1, and PF13, for ciliary dynein preassembly. Based on analysis in single and double PIH mutants, specific PIH proteins are required for assembly of specific dyneins, in some cases, and in other cases the PIH proteins can work in partially overlapping fashion. Further biochemical studies, and the two novel *Chlamydomonas* PIH preassembly mutants, *mot48-2* and *twi1-1*, from this study, will define our understanding of dynein assembly.

## Materials and methods

### *Chlamydomonas* strains and samples

The *Chlamydomonas* strains used in this study are listed in S2 Table. The novel *mot48* (*ida10*) allele, referred to as *mot48-2* was isolated from a CLiP library strain (LMJ.RY0402.055540)[24] having a mutation that was unlinked to the paromomycin resistant (*APHVIII*) insertional cassette used for selection but caused a slow-swimming phenotype. The original LMJ.RY0402.055540 strain was backcrossed with the wild-type (CC-125) strain to separate this mutation, and two *mot48-2* progeny were isolated. The identification of the *mot48* background was confirmed by Sanger sequencing using the primer pair: Ida10-2 GF2 (5’-TGGCAGCACATTCATAAGCA-3’) and Ida10-2 GR2 (5’-CGCTGTACTAGAGCCCCTCA-3’). The *twi1* strain was first obtained from the CLiP library (LMJ.RY0402.076787)[24] and backcrossed with the wild-type strain (CC-125), and the *twi1* mutant progeny (*twi1-1*) were used for experiments. Cells were grown in the tris-acetic acid-phosphate (TAP) liquid/solid media as previously described [45]. Double *Chlamydomonas* PIH mutants were obtained using the standard tetrad procedure [45]. The deciliation was performed following the standard procedure [46]. For preparation of whole cell samples or de-ciliated cell-body samples, whole cells/cell bodies were extracted with water/methanol/chloroform (volume ratio = 3:4:1) to remove the nucleic acids, lipids and chlorophyll, and the denatured proteins were boiled in the SDS-sample buffer as previously described [47].

### Rescue of *mot48-2*, *twi1-1*, and *mot48-2; twi1-1*

Phenotypic rescue of the *mot48-2*, *twi1-1*, and *mot48-2; twi1-1* strains was performed by the electroporation method using each wild-type gene cloned in the modified pGend vector (pGend-MCS-3HA-AphVIII/pGend-MCS-mCherry-3HA-AphVIII/pGend-MCS-3HA-Hyg)[13, 48, 49]. The rescued strain, *mot48-2; MOT48*::*HA* expressed exogeneous MOT48 with a 3HA tag at the C-terminus. The rescued strain, *mot48-2; MOT48*::*mCherry-HA* expressed exogeneous MOT48 with the mCherry-3HA tag at the C-terminus. The pGend-MOT48-3HA-AphVIII vector was previously described [13] and also used in this study. The two *twi1-1* rescued strains, *twi1-1; TWI1*::*HA* and *mot48-2; twi1-1; TWI1*::*HA* expressed exogeneous TWI1 with a 3HA tag at the C-terminus. The primer pair used for wild-type *TWI1* cloning was as follows, TWI1-pGend-F1 (5’-CACAACAAGCCCATATGGACATTGGGAGCTTCACTGCTGA-3’) and TWI1-pGend-R1 (5’-GGTATCGATCGAATTCGAATGGCTCTTCCCGAATGATGCG-3’). The NdeI/EcoRI sites used for cloning are underlined.

### Spectral counting analysis

A semi-quantitative estimation of the amount of dyneins in the isolated axonemes [28, 50, 51] was conducted using the spectral counting analyses on some LC-MS/MS spectrometers at University of Massachusetts Medical School Mass Spectrometry Facility.

A first set of experiments was performed on dyneins from the wild-type (CC-124, *cw15*), *mot48-1*, and *mot48-2* strains with the aim of comparing the dynein levels between the *mot48-1* and *mot48-2* strains. For normalization and comparison between samples, the peptide numbers of ciliary dyneins observed in the CC-124 strain were assigned a ratio of 1.0, and the observed peptide numbers of Hydin [52], a central-pair protein were also used as an internal standard. The first-set of experiments was generously performed by Dr. John Leszyk (University of Massachusetts Medical School). Thresholds in the Scaffold 4 software (http://www.proteomesoftware.com/products/scaffold/) for the first-set of analyses were set as follows: Protein Threshold: 90%/Minimal Peptides Number: 2/ Peptide Threshold: 70%. The averages of two independent experiments are summarized in Fig 2.

A second set of experiments was performed on dyneins from wild-type (CC-125), *twi1-1*, *mot48-2*, *mot48-2; twi1-1*, *pf13*, *pf13; twi1-1*, and *pf23* (5–4) strains with the aim of comparing the dynein levels between the single and double PIH preassembly mutants. For normalization and comparison between samples, observed peptide numbers in the CC-125 strain and also the peptide numbers of Hydin were used. The second set of experiments was generously performed by Drs. Scott Shaffer and Xuni Li (University of Massachusetts Medical School). Thresholds in the Scaffold 4 software for the second-set analyses were set as follows: Protein Threshold: 99%/Minimal Peptides Number: 2/ Peptide Threshold: 70%. The results are summarized in Fig 4 and S3 Fig.

A third set of experiments was performed on dyneins from wild-type (CC-125), *twi1-1; TWI1*::*HA*, *pf22* (CC-1382 with the *twi1-2* background) and *pf22A* (CC-2493) strains with the aim to check the dynein rescue and effect of the *twi1-2* mutation in dynein assembly in *pf22* strains. Observed peptide numbers were normalized using peptide numbers of CC-125 and Hydin, and the results were combined/incorporated into Fig 4 and S3 Fig. The third set of experiments was generously performed by Drs. Scott Shaffer and Roshanak Aslebagh (University of Massachusetts Medical School). Thresholds in the Scaffold 4 software for the third-set analyses were set as follows: Protein Threshold: 99%/Minimal Peptides Number: 2/ Peptide Threshold: 77%. The spectral counting data of *pf23* (CC-1383 with the *twi1-2* background) normalized with the wild-type (137c) peptide counts were reanalyzed/refined from our previous paper [28] with the Hydin normalization in S3 Fig.

### TWI1 antibody production

The TWI1 cDNA sequence was determined using the *Chlamydomonas* cDNA library (*Chlamydomonas* Resource Center) and cloned into the NdeI/BamHI site of the pET15b vector (Novagen) by the In-Fusion HD Cloning enzyme (TAKARA). The primer pair to amplify the *TWI1* cDNA sequence was as follows: TWI1-CF1 (5’-CGCGCGGCAGCCATATGGACATTGGGAGCTTCACTG-3’) and TWI1-CR1 (5’-GTTAGCAGCCGGATCCGAATGGCTCTTCCCGAATGATGCG-3’)(The NdeI/BamHI sites are underlined). The purified TWI1 protein with a 6His tag at the N-terminus was used as antigen to immunize two rabbits. The antisera from rabbits were blot and Protein-A purified before use, as previously described [53, 54]. The *Chlamydomonas TWI1* cDNA sequence determined in this study was deposited in the DNA Data Bank of Japan (DDBJ) under the accession No. LC461993.

### Other methods

SDS-PAGE and immunoblotting were performed following standard procedures [55, 56]. For the immunoblotting, antibodies used included: primary antibodies (anti-MOT48 [28], anti-HA (Y-11)(Santa Cruz), anti-HA (3F10)-HRP (Roche), anti-PF23 [28], anti-TWI1 (this study), anti-actin/IDA5 [57], anti-p28/IDA4 [58], anti-centrin/VFL2 (20H5)(MilliporeSigma), anti-p38 [59], anti-IC138/BOP5 [60], anti-IC2/IC69/ODA6 [61], anti-DHC9 (IDA c HC)[37]); secondary antibody (Goat-anti-Rabbit or Mouse-HRP (Roche)). Immunofluorescent microscopic observation of nucleo-flagellar apparatuses was performed as described previously [36, 37, 62], and the acquired images were adjusted for presentation using Photoshop (Adobe). The urea PAGE used to resolve ciliary dynein bands was performed as previously described [63]. The swimming velocity of *Chlamydomonas* was assessed on free-swimming cells in liquid culture using our in-lab video system and ImageJ software (https://www.google.com/search?client=firefox-b-d&q=imagej+software) [64]. Velocities were measured on the 3-day liquid cultured cells, and reported in the text as average ± standard deviation. The ciliated cell ratio in the *pf13* and *pf13; twi1-1* strains was counted and averaged on three days. Student’s t-test was performed on Excel (Microsoft).

## Supporting information

S1 Fig Swimming velocity measurement of *mot48-2/twi1-1*-related strains. Swimming velocities of wild-type (CC-125), *mot48-2*, *mot48-2; MOT48*::*HA*, *mot48-2; MOT48*::*mCherry-HA*, *twi1-1*, *twi1-1; TWI1*::*HA*, *mot48-2; twi1-1*, and *mot48-2; twi1-1; TWI1*::*HA*. For wild-type (CC-125), *twi1-1*, *twi1-1; TWI1*::*HA*, *mot48-2; MOT48*::*HA*, and *mot48-2; MOT48*::*mCherry-HA*, more than 40 cells were measured. For *mot48-2*, *mot48-2; twi1-1*, and *mot48-2; twi1-1; TWI1*::*HA*, it was difficult to find ideal cells for the velocity measurement, but more than 15 cells were measured. As discussed in the main text, the swimming phenotypes of the preassembly mutants slightly varied from day to day and culture to culture because of the apparent compensatory and overlapping nature of the dynein preassembly. In this figure, swimming velocities are shown for cells cultured for 3 days in the liquid TAP media in mini petri-dishes under constant light. Asterisks indicate p < 0.01 in the Student’s t-test.
(TIF)

S2 Fig Immunofluorescent microscopic observation of ciliary dyneins in axonemes from PIH mutants. Immunofluorescence localization of DHC9 (IDA c HC), DHC11 (minor dynein HC) and α-tubulin in wild-type (CC-124), *pf13* and *mot48-2* nucleo-flagellar apparatuses. DHC11 was shown to be localized at the proximal part of the wild-type axonemes [37]. Both DHC9 and DHC11 signals were reduced in the *pf13* and *mot48-2* axonemes compared to wild-type axonemes. The bright puncta are non-specific staining/autofluorescence. Bar: ~ 5 μm.
(TIF)

S3 Fig Dynein defects are more severe in the *pf23* mutant also containing the *twi1-2* mutation. **A)** Spectral counting comparison of dyneins from axonemes of *pf23* (5–4), *pf23* (CC-1383; with the *twi1-2* background), *pf22A* (CC-2493), and *pf22* (CC-1382; with the *twi1-2* background). The spectral data of *pf23* (5–4) are from the second set of experiments. The spectral data of *pf22A* (CC-2493) and *pf22* (CC-1382) are from the third set of experiments. The spectral data of *pf23* (CC-1383) are refined/reanalyzed from our previous study [28]. The spectral numbers observed in the mutants were normalized using the spectral numbers of Hydin and wild-type peptides (CC-125 for *pf23* (5–4), *pf22A* (CC-2493), and *pf22* (CC-1382), and 137c for *pf23* (CC-1383)[28]). Asterisks indicate the ciliary dynein species for which the spectral numbers in the *pf23* strain (CC-1383; with the *twi1-2* background) showed more than a 50% reduction compared to the *pf23* (5–4) strain. **B)** Ciliary dynein species for which the spectral numbers in the *pf23* (5–4) or *pf22A* (CC-2493) strain (without the *twi1-2* background) showed a more than 50% reduction compared to wild-type (CC-125) are summarized.
(TIF)

S4 Fig Phylogenetic analysis of PIH proteins from *Chlamydomonas reinhardtii* and other organisms. The protein alignment was performed using the ClustalW software (v2.1)(http://clustalw.ddbj.nig.ac.jp/) by the default settings, and the phylogenetic tree was drawn by the Neighbor-Joining method [72] and modified in MEGA7 program (https://www.megasoftware.net/). The bootstrap consensus tree inferred from 1000 replicates is shown, and the bootstrap numbers are shown in percentile [73]. The evolutionary distances were computed using the p-distance method [74], and all positions containing gaps and missing data were eliminated. The DNAAF4/DYX1C1/PF23 proteins, which have the CS (CHORD-containing proteins and SGT1) domain relating to the PIH1 domain [18] were used as an outgroup. In this tree, *Chlamydomonas* MOT48 falls into the PIH1D1 group. The accession numbers of proteins used to draw this tree were as follows: Human DNAAF2/KTU (NCBI: ACN30493.1); Mouse DNAAF2/KTU (NCBI: NP_081545.3); Zebrafish KTU (NCBI: NP_001028272.1); *Chlamydomonas* PF13 (NCBI: BAG69288.1); Human PIH1D1 (NCBI: NP_060386.1); Mouse PIH1D1 (NCBI: AAH68254.1); Zebrafish PIH1D1 (NCBI: NP_001153400.1); Human PIH1D2 (NCBI: AAH19238.1); Mouse PIH1D2 (NCBI: AAH39645.1); Zebrafish PIH1D2 (NCBI: NP_001008629.1); *Chlamydomonas* MOT48 (NCBI: BAI83444.1); Human DNAAF6/PIH1D3 (NCBI: NP_001162625.1); Mouse DNAAF6a/PIH1D3a (NCBI: NP_083338.1); Mouse DNAAF6b/PIH1D3b/Twister2 (NCBI: AAI19079.1)[21]; Zebrafish Twister (NCBI: NP_001002309.1); *Chlamydomonas* TWI1 (NCBI: LC461993, This study); Human DNAAF4/DYX1C1 (NCBI: NP_570722.2); Mouse DNAAF4/DYX1C1 (NCBI: NP_080590.3); Zebrafish DNAAF4/DYX1C1 (NCBI: NP_991251.1); *Chlamydomonas* PF23 (NCBI: BBA27223.1); Yeast Nop17 (NCBI: GAX71541.1).
(TIF)

S1 Table *Chlamydomonas* dynein subunits, IFT proteins, and non-PIH preassembly proteins mentioned in this study and their potential human orthologues. (PDF)

S2 Table *Chlamydomonas* strains used in this study. (PDF)
