# Supplementary figures and images for: Mutations in PIH proteins MOT48, TWI1 and PF13 define common and unique steps for preassembly of each, different ciliary dynein
Source: PLoS Genet. 2020 Nov 3;16(11):e1009126. doi: 10.1371/journal.pgen.1009126 (PMC7608865; doi:10.1371/journal.pgen.1009126)

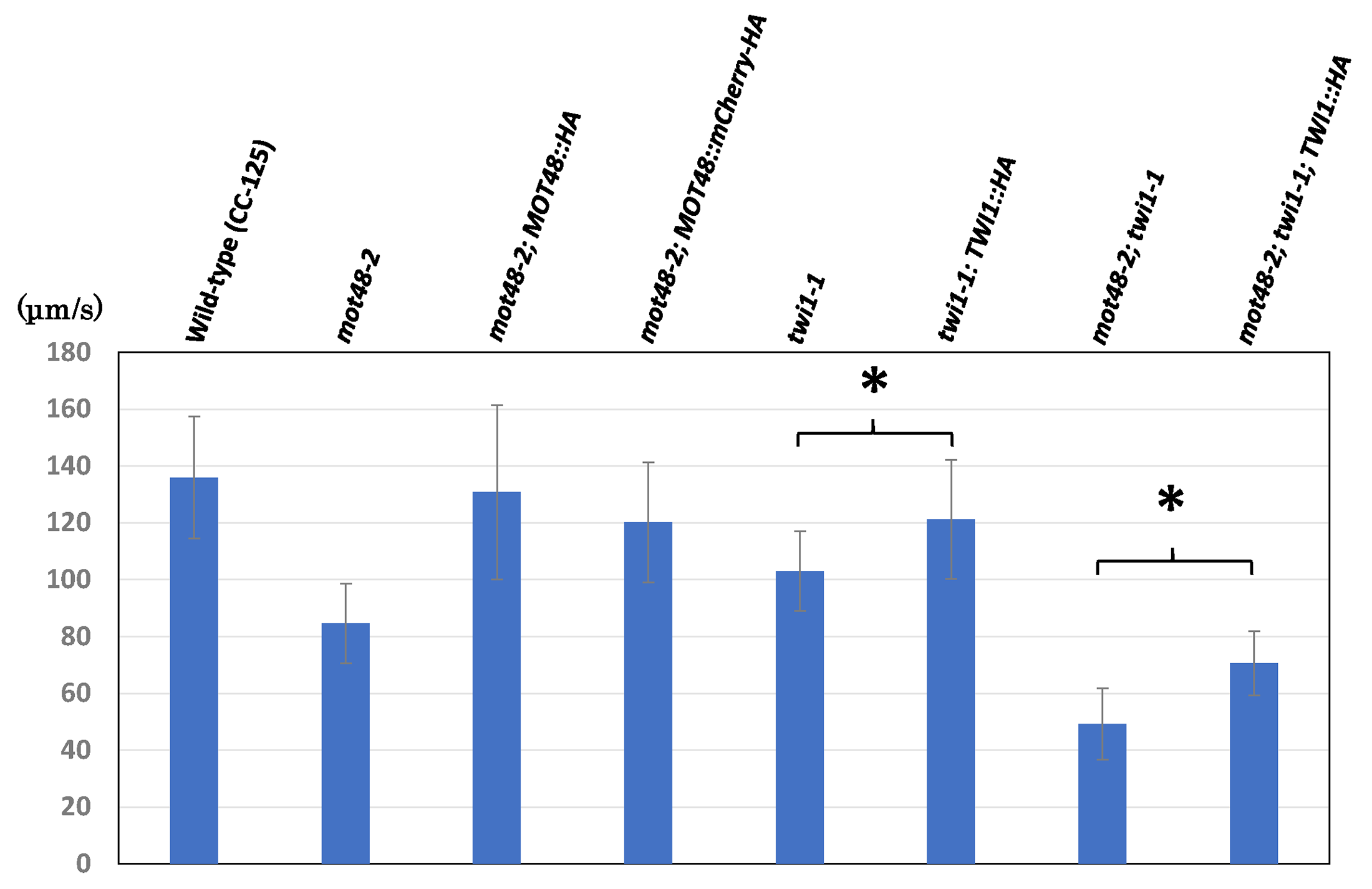

Supplement: S1 Fig — Swimming velocities of wild-type (CC-125), mot48-2, mot48-2; MOT48::HA, mot48-2; MOT48::mCherry-HA, twi1-1, twi1-1; TWI1::HA, mot48-2; twi1-1, and mot48-2; twi1-1; TWI1::HA. For wild-type (CC-125), twi1-1, twi1-1; TWI1::HA, mot48-2; MOT48::HA, and mot48-2; MOT48::mCherry-HA, more than 40 cells were measured. For mot48-2, mot48-2; twi1-1, and mot48-2; twi1-1; TWI1::HA, it was difficult to find ideal cells for the velocity measurement, but more than 15 cells were measured. As discussed in the main text, the swimming phenotypes of the preassembly mutants slightly varied from day to day and culture to culture because of the apparent compensatory and overlapping nature of the dynein preassembly. In this figure, swimming velocities are shown for cells cultured for 3 days in the liquid TAP media in mini petri-dishes under constant light. Asterisks indicate p < 0.01 in the Student’s t-test. (TIF) [file pgen.1009126.s001.tif]

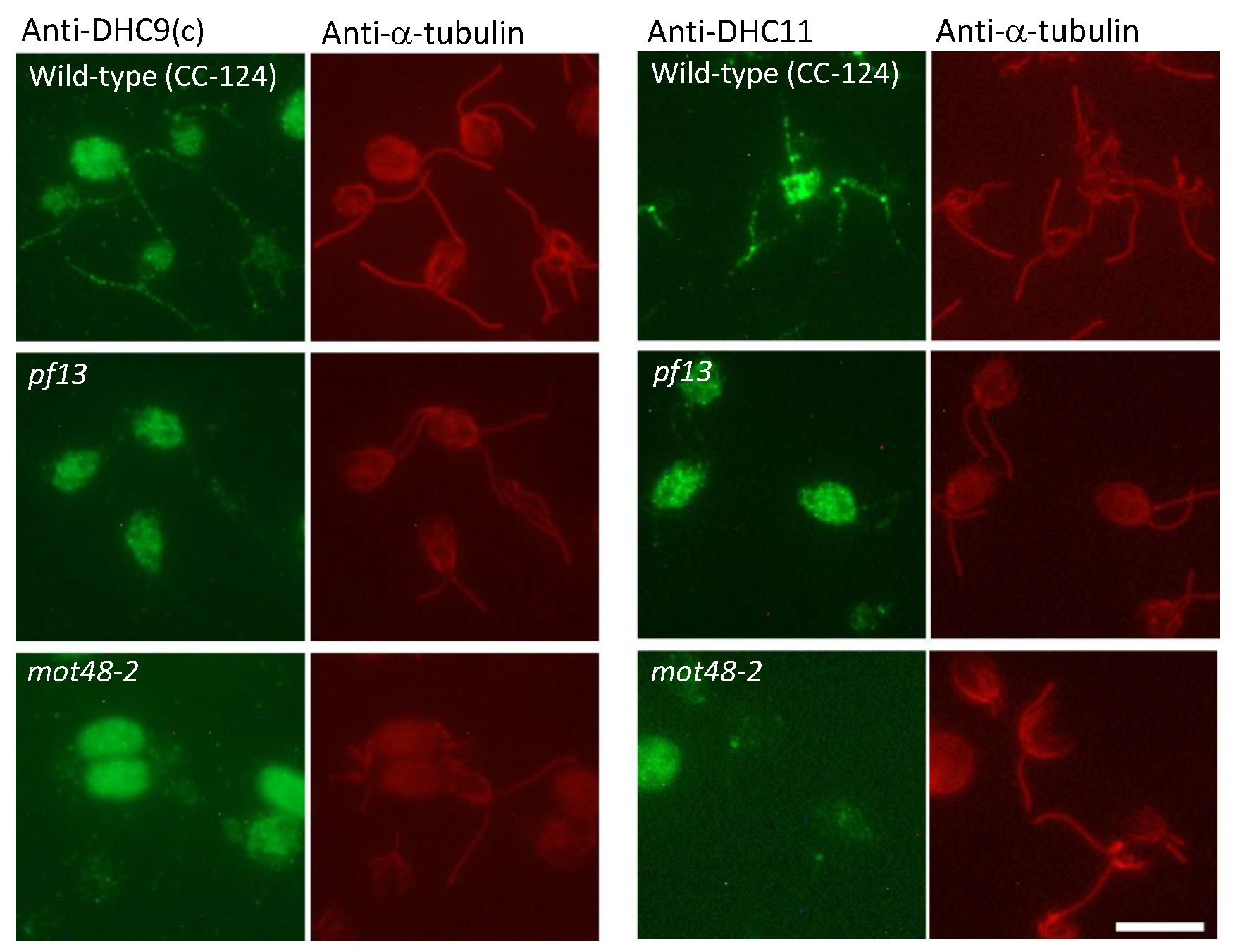

Supplement: S2 Fig — Immunofluorescence localization of DHC9 (IDA c HC), DHC11 (minor dynein HC) and α-tubulin in wild-type (CC-124), pf13 and mot48-2 nucleo-flagellar apparatuses. DHC11 was shown to be localized at the proximal part of the wild-type axonemes [37]. Both DHC9 and DHC11 signals were reduced in the pf13 and mot48-2 axonemes compared to wild-type axonemes. The bright puncta are non-specific staining/autofluorescence. Bar: ~ 5 μm. (TIF) [file pgen.1009126.s002.tif]

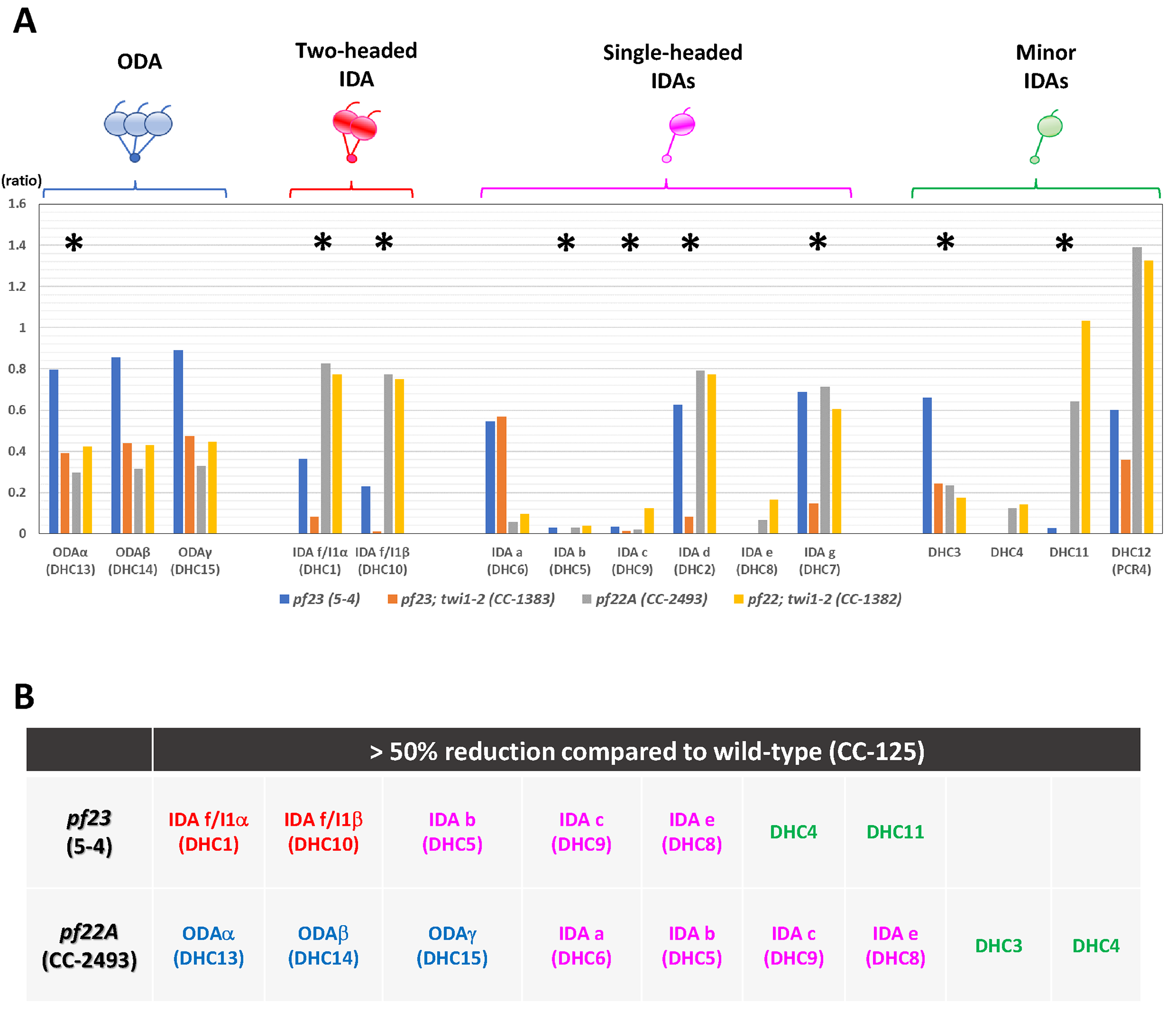

Supplement: S3 Fig — A) Spectral counting comparison of dyneins from axonemes of pf23 (5–4), pf23 (CC-1383; with the twi1-2 background), pf22A (CC-2493), and pf22 (CC-1382; with the twi1-2 background). The spectral data of pf23 (5–4) are from the second set of experiments. The spectral data of pf22A (CC-2493) and pf22 (CC-1382) are from the third set of experiments. The spectral data of pf23 (CC-1383) are refined/reanalyzed from our previous study [28]. The spectral numbers observed in the mutants were normalized using the spectral numbers of Hydin and wild-type peptides (CC-125 for pf23 (5–4), pf22A (CC-2493), and pf22 (CC-1382), and 137c for pf23 (CC-1383)[28]). Asterisks indicate the ciliary dynein species for which the spectral numbers in the pf23 strain (CC-1383; with the twi1-2 background) showed more than a 50% reduction compared to the pf23 (5–4) strain. B) Ciliary dynein species for which the spectral numbers in the pf23 (5–4) or pf22A (CC-2493) strain (without the twi1-2 background) showed a more than 50% reduction compared to wild-type (CC-125) are summarized. (TIF) [file pgen.1009126.s003.tif]

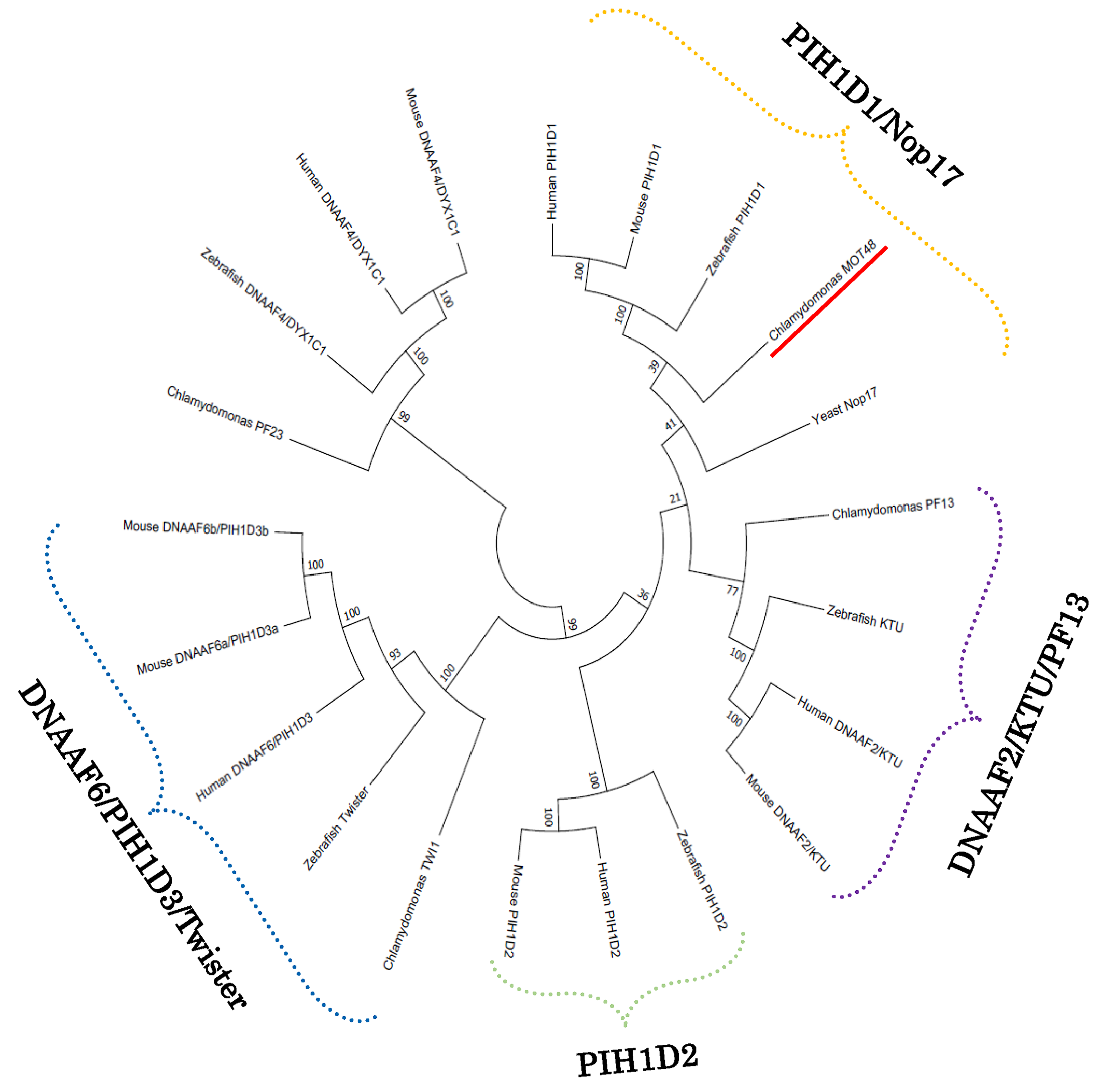

Supplement: S4 Fig — The protein alignment was performed using the ClustalW software (v2.1)(http://clustalw.ddbj.nig.ac.jp/) by the default settings, and the phylogenetic tree was drawn by the Neighbor-Joining method [72] and modified in MEGA7 program (https://www.megasoftware.net/). The bootstrap consensus tree inferred from 1000 replicates is shown, and the bootstrap numbers are shown in percentile [73]. The evolutionary distances were computed using the p-distance method [74], and all positions containing gaps and missing data were eliminated. The DNAAF4/DYX1C1/PF23 proteins, which have the CS (CHORD-containing proteins and SGT1) domain relating to the PIH1 domain [18] were used as an outgroup. In this tree, Chlamydomonas MOT48 falls into the PIH1D1 group. The accession numbers of proteins used to draw this tree were as follows: Human DNAAF2/KTU (NCBI: ACN30493.1); Mouse DNAAF2/KTU (NCBI: NP_081545.3); Zebrafish KTU (NCBI: NP_001028272.1); Chlamydomonas PF13 (NCBI: BAG69288.1); Human PIH1D1 (NCBI: NP_060386.1); Mouse PIH1D1 (NCBI: AAH68254.1); Zebrafish PIH1D1 (NCBI: NP_001153400.1); Human PIH1D2 (NCBI: AAH19238.1); Mouse PIH1D2 (NCBI: AAH39645.1); Zebrafish PIH1D2 (NCBI: NP_001008629.1); Chlamydomonas MOT48 (NCBI: BAI83444.1); Human DNAAF6/PIH1D3 (NCBI: NP_001162625.1); Mouse DNAAF6a/PIH1D3a (NCBI: NP_083338.1); Mouse DNAAF6b/PIH1D3b/Twister2 (NCBI: AAI19079.1)[21]; Zebrafish Twister (NCBI: NP_001002309.1); Chlamydomonas TWI1 (NCBI: LC461993, This study); Human DNAAF4/DYX1C1 (NCBI: NP_570722.2); Mouse DNAAF4/DYX1C1 (NCBI: NP_080590.3); Zebrafish DNAAF4/DYX1C1 (NCBI: NP_991251.1); Chlamydomonas PF23 (NCBI: BBA27223.1); Yeast Nop17 (NCBI: GAX71541.1). (TIF) [file pgen.1009126.s004.tif]
