# Supplementary material for: Mutations in PIH proteins MOT48, TWI1 and PF13 define common and unique steps for preassembly of each, different ciliary dynein
Source: PLoS Genet. 2020 Nov 3;16(11):e1009126. doi: 10.1371/journal.pgen.1009126 (PMC7608865; doi:10.1371/journal.pgen.1009126)
Supplement: S1 Table — (PDF) [file pgen.1009126.s005.pdf]

Supplemental Table 1. *Chlamydomonas* dynein subunits, IFT proteins, and non-PIH preassembly proteins mentioned in this study and their potential human orthologues

Chlamydomonas Protein Name	Human Orthologue	Reference
**Dynein subunits** (species)		
**HCs**		
DHC1 (IDA f/I1α)	DNAH10	[1, 2]
DHC2 (IDA d)	DNAH1	[1, 2]
DHC3 (Minor dynein)	DNAH14	[2]
DHC4 (Minor dynein)	N/A	[2]
DHC5 (IDA b)	DNAH3	[1, 2]
DHC6 (IDA a)	DNAH7	[1, 2]
DHC7 (IDA g)	DNAH6	[2]
DHC8 (IDA e)	N/A	[2]
DHC9 (IDA c)	DNAH12	[1, 2]
DHC10 (IDA f/I1β)	DNAH2	[1, 2]
DHC11 (Minor dynein)	N/A	[2]
DHC12/PCR4 (Minor dynein)	N/A	[1, 2]
DHC13 (ODAα)	N/A	[1, 2]
DHC14 (ODAβ)	DNAH9/DNAH11/DNAH17	[1-3]
DHC15 (ODAγ)	DNAH5/DNAH8	[1, 3]
**IC/LCs** (species)		
Actin/IDA5 (single-headed IDAs)	Actin	[1, 4]
Centrin/VFL2 (IDAs b/e/g and DHC3/DHC4)	CENT1/CENT2/CENT3	[1, 5]
IC2/IC69/ODA6 (ODAs)	DNAI2	[1, 6]
IC138/BOP5 (IDA f/I1)	WDR78	[1, 7]
p28/IDA4 (IDAs a/c/d and DHC11)	DNALI1	[1, 5, 8]
p38 (IDA d)	ZMYND12	[1, 9]
**IFT proteins**		
IFT46	IFT46/C11ORF60	[10, 11]
IFT74	IFT74	[10, 11]
**Non-PIH preassembly proteins**		
FBB18	C21ORF59	[12]
ODA5	CCDC63	[1, 13]
ODA7	DNAAF1/LRRC50	[1, 14]
ODA8	LRRC56	[15, 16]
ODA10	CCDC151	[17, 18]

ODA16	WDR69	[1, 19]
PF22	DNAAF3	[1, 20]
PF23	DNAAF4/DYX1C1	[21, 22]

References

1. Hom EF, Witman GB, Harris EH, Dutcher SK, Kamiya R, Mitchell DR, et al. A unified taxonomy for ciliary dyneins. Cytoskeleton (Hoboken). 2011;68(10):555-65.

2. Kollmar M. Fine-Tuning Motile Cilia and Flagella: Evolution of the Dynein Motor Proteins from Plants to Humans at High Resolution. Mol Biol Evol. 2016;33(12):3249-67.

3. Inaba K, Mizuno K. Sperm dysfunction and ciliopathy. Reprod Med Biol. 2016;15(2):77-94.

4. Piperno G, Luck DJ. An actin-like protein is a component of axonemes from Chlamydomonas flagella. J Biol Chem. 1979;254(7):2187-90.

5. Yagi T, Uematsu K, Liu Z, Kamiya R. Identification of dyneins that localize exclusively to the proximal portion of Chlamydomonas flagella. J Cell Sci. 2009;122(9):1306-14.

6. Pennarun G, Chapelin C, Escudier E, Bridoux AM, Dastot F, Cacheux V, et al. The human dynein intermediate chain 2 gene (DNAI2): cloning, mapping, expression pattern, and evaluation as a candidate for primary ciliary dyskinesia. Hum Genet. 2000;107(6):642-9.

7. Zhang Y, Chen Y, Zheng J, Wang J, Duan S, Zhang W, et al. Vertebrate Dynein-f depends on Wdr78 for axonemal localization and is essential for ciliary beat. J Mol Cell Biol. 2019;11(5):383-94.

8. Rashid S, Breckle R, Hupe M, Geisler S, Doerwald N, Neesen J. The murine Dnali1 gene encodes a flagellar protein that interacts with the cytoplasmic dynein heavy chain 1. Mol Reprod Dev. 2006;73(6):784-94.

9. Yamamoto R, Yanagisawa HA, Yagi T, Kamiya R. A novel subunit of axonemal dynein conserved among lower and higher eukaryotes. FEBS Lett. 2006;580(27):6357-60.

10. Taschner M, Lorentzen E. The Intraflagellar Transport Machinery. Cold Spring Harb Perspect Biol. 2016;8(10):a028092.

11. Pedersen LB, Rosenbaum JL. Intraflagellar transport (IFT) role in ciliary assembly, resorption and signalling. Curr Top Dev Biol. 2008;85:23-61.

12. Austin-Tse C, Halbritter J, Zariwala MA, Gilberti RM, Gee HY, Hellman N, et al. Zebrafish Ciliopathy Screen Plus Human Mutational Analysis Identifies C21orf59 and CCDC65 Defects as Causing Primary Ciliary Dyskinesia. Am J Hum Genet. 2013;93(4):672-86.

13. Wirschell M, Pazour G, Yoda A, Hirono M, Kamiya R, Witman GB. Oda5p, a novel axonemal protein required for assembly of the outer dynein arm and an associated adenylate kinase. Mol Biol Cell. 2004;15(6):2729-41.

14. Duquesnoy P, Escudier E, Vincensini L, Freshour J, Bridoux AM, Coste A, et al.

Loss-of-function mutations in the human ortholog of Chlamydomonas reinhardtii ODA7 disrupt dynein arm assembly and cause primary ciliary dyskinesia. Am J Hum Genet. 2009;85(6):890-6.

15. Desai PB, Freshour JR, Mitchell DR. Chlamydomonas axonemal dynein assembly locus ODA8 encodes a conserved flagellar protein needed for cytoplasmic maturation of outer dynein arm complexes. Cytoskeleton (Hoboken). 2015;72(1):16-28.

16. Bonnefoy S, Watson CM, Kernohan KD, Lemos M, Hutchinson S, Poulter JA, et al. Biallelic Mutations in LRRC56, Encoding a Protein Associated with Intraflagellar Transport, Cause Mucociliary Clearance and Laterality Defects. Am J Hum Genet. 2018;103(5):727-39.

17. Dean AB, Mitchell DR. Chlamydomonas ODA10 is a conserved axonemal protein that plays a unique role in outer dynein arm assembly. Mol Biol Cell. 2013;24(23):3689-96.

18. Alsaadi MM, Erzurumluoglu AM, Rodriguez S, Guthrie PA, Gaunt TR, Omar HZ, et al. Nonsense mutation in coiled-coil domain containing 151 gene (CCDC151) causes primary ciliary dyskinesia. Hum Mutat. 2014;35(12):1446-8.

19. Gao C, Wang G, Amack JD, Mitchell DR. Oda16/Wdr69 is essential for axonemal dynein assembly and ciliary motility during zebrafish embryogenesis. Dev Dyn. 2010;239(8):2190-7.

20. Mitchison HM, Schmidts M, Loges NT, Freshour J, Dritsoula A, Hirst RA, et al. Mutations in axonemal dynein assembly factor DNAAF3 cause primary ciliary dyskinesia. Nat Genet. 2012;44(4):381-9, s1-2.

21. Tarkar A, Loges NT, Slagle CE, Francis R, Dougherty GW, Tamayo JV, et al. DYX1C1 is required for axonemal dynein assembly and ciliary motility. Nat Genet. 2013;45(9):995-1003.

22. Yamamoto R, Obbineni JM, Alford LM, Ide T, Owa M, Hwang J, et al. Chlamydomonas DYX1C1/PF23 is essential for axonemal assembly and proper morphology of inner dynein arms. PLoS Genet. 2017;13(9):e1006996.
