# Supplementary material for: Mutations in PIH proteins MOT48, TWI1 and PF13 define common and unique steps for preassembly of each, different ciliary dynein
Source: PLoS Genet. 2020 Nov 3;16(11):e1009126. doi: 10.1371/journal.pgen.1009126 (PMC7608865; doi:10.1371/journal.pgen.1009126)
Supplement: S2 Table — (PDF) [file pgen.1009126.s006.pdf]

Supplemental Table 2. *Chlamydomonas* strains used in this study

Strain Name	Responsible Protein/Description	Axonemal Defect	Reference
CC-124	N/A	Normal	[1]
CC-125	N/A	Normal	[1]
cw15 (CC-4533)	Likely cell-wall protein, regarded as wild-type in this study	Normal	[2, 3]
fbb18	FBB18	Defects in both ODA and IDAs	Manuscript in preparation
ift46-1 (CC-4375)	IFT46	Paralyzed short cilia, defects in ODA and central-pair	[4, 5]
ift74-1 (CC-5159)	IFT74	Very short or no cilia	[6]
ift74-2 (CC-5161)	IFT74	No cilia	[6]
mot48-1 (*ida10-1*)	MOT48/IDA10	Slight defects in ODA, reduced amounts of some IDAs	[7, 8]
mot48-2 (*ida10-2*)	MOT48/IDA10; a new allele of *mot48*	Slight defects in ODA, reduced amounts of some IDAs	This study, [9]
mot48-2; twi1-1	MOT48/IDA10 and TWI1; PIH double mutants	Slight defects in ODA, greatly reduced amounts of some IDAs, severer than *mot48-2*	This study
mot48-2; twi1-1; TWI1::HA	*mot48-2; twi1-1* strain rescued with 3HA tagged TWI1	Slight defects in ODA, reduced amounts of some IDAs, apparently similar to *mot48-2*	This study
mot48-2; pf13	MOT48/IDA10 and PF13; PIH double mutants	Very short or no cilia, dynein defect not analyzable	This study
mot48-2; MOT48::HA	*mot48-2* strain rescued with 3HA tagged MOT48	Not analyzed	This study
mot48-2; MOT48::mCherry-HA	*mot48-2* strain rescued with mCherry-3HA tagged MOT48	Not analyzed	This study
oda5 (CC-2236)	ODA5	Lacking ODA	[8, 10, 11]
oda7 (CC-2240)	ODA7	Lacking ODA	[8, 10, 12]
oda8 (CC-2242)	ODA8	Lacking ODA	[8, 10, 13]
oda10 (CC-2246)	ODA10	Lacking ODA	[10, 14]
oda11 (CC-2672)	ODA11/DHC13	Lacking HCα of ODA	[10, 15]
oda16-1 (CC-4554)	ODA16	Lacking ODA	[8, 16]

pf13	PF13	Greatly reduced ODA and reduced amounts of some IDAs	[8, 17, 18]
pf13; twi1-1	PF13 and TWI1; PIH double mutants	Greatly reduced ODA and some IDAs, severer than *pf13*	This study
pf22 **(CC-1382)**	PF22 and TWI1; found to have *twi1-2* background in this study	Lacking ODA and reduced amounts of some IDAs	[17, 19]
pf22 **(CC-2495)**	PF22	Lacking ODA and reduced amounts of some IDAs	[8, 17, 19]
pf22A **(CC-2493)**	PF22	Lacking ODA and reduced amounts of some IDAs	[8, 17, 19]
pf23 **(CC-1383)**	PF23 and TWI1; found to have *twi1-2* background in this study	Slight defects in ODA, greatly reduced amounts of IDAs	[17, 20]
pf23 **(CC-3660)**	PF23 and TWI1; found to have *twi1-2* background in this study	Slight defects in ODA, greatly reduced amounts of IDAs	[17, 20]
pf23 **(5-4)**	PF23; parent strain used to map the *PF23/DYX1C1* locus and for part of the rescue experiments in our previous study [20]	Subtle defects in ODA, greatly reduced amounts of some IDAs	[17, 20]
twi1-1	TWI1; progeny from mating cross of wild-type (CC-125) and the LMJ.RY0402.076787 CLiP strain	Slight defects in IDAs "c, e"	This study, [9]
twi1-1; TWI1::HA	*twi1-1* strain rescued with 3HA tagged TWI1	Apparently normal	This study

References

1. Harris EH. The Chlamydomonas sourcebook: a comprehensive guide to biology and laboratory use: Academic Press, San Diego, 780pp; 1989.

2. Davies DR, Plaskitt A. Genetical and structural analyses of cell-wall formation in Chlamydomonas reinhardi. Genet Res. 1971;17(1):33-43.

3. Hyams J, Davies DR. The induction and characterisation of cell wall mutants of Chlamydomonas reinhardi. Mutat Res. 1972;14(4):381-89.

4. Lv B, Wan L, Taschner M, Cheng X, Lorentzen E, Huang K. Intraflagellar transport protein IFT52 recruits IFT46 to the basal body and flagella. J Cell Sci. 2017;130(9):1662-74.

5. Hou Y, Qin H, Follit JA, Pazour GJ, Rosenbaum JL, Witman GB. Functional analysis of an individual IFT protein: IFT46 is required for transport of outer dynein arms into flagella. J Cell Biol. 2007;176(5):653-65.

6. Brown JM, Cochran DA, Craige B, Kubo T, Witman GB. Assembly of IFT trains at

the ciliary base depends on IFT74. Curr Biol. 2015;25(12):1583-93.

7. Yamamoto R, Hirono M, Kamiya R. Discrete PIH proteins function in the cytoplasmic preassembly of different subsets of axonemal dyneins. J Cell Biol. 2010;190(1):65-71.

8. Hom EF, Witman GB, Harris EH, Dutcher SK, Kamiya R, Mitchell DR, et al. A unified taxonomy for ciliary dyneins. Cytoskeleton (Hoboken). 2011;68(10):555-65.

9. Li X, Zhang R, Patena W, Gang SS, Blum SR, Ivanova N, et al. An indexed, mapped mutant library enables reverse genetics studies of biological processes in Chlamydomonas reinhardtii. Plant Cell. 2016;28(2):367-87.

10. Kamiya R. Mutations at twelve independent loci result in absence of outer dynein arms in Chylamydomonas reinhardtii. J Cell Biol. 1988;107(6):2253-8.

11. Wirschell M, Pazour G, Yoda A, Hirono M, Kamiya R, Witman GB. Oda5p, a novel axonemal protein required for assembly of the outer dynein arm and an associated adenylate kinase. Mol Biol Cell. 2004;15(6):2729-41.

12. Freshour J, Yokoyama R, Mitchell DR. Chlamydomonas flagellar outer row dynein assembly protein ODA7 interacts with both outer row and I1 inner row dyneins. J Biol Chem. 2007;282(8):5404-12.

13. Desai PB, Freshour JR, Mitchell DR. Chlamydomonas axonemal dynein assembly locus ODA8 encodes a conserved flagellar protein needed for cytoplasmic maturation of outer dynein arm complexes. Cytoskeleton (Hoboken). 2015;72(1):16-28.

14. Dean AB, Mitchell DR. Chlamydomonas ODA10 is a conserved axonemal protein that plays a unique role in outer dynein arm assembly. Mol Biol Cell. 2013;24(23):3689-96.

15. Sakakibara H, Mitchell DR, Kamiya R. A Chlamydomonas outer arm dynein mutant missing the alpha heavy chain. J Cell Biol. 1991;113(3):615-22.

16. Ahmed NT, Mitchell DR. ODA16p, a Chlamydomonas flagellar protein needed for dynein assembly. Mol Biol Cell. 2005;16(10):5004-12.

17. Huang B, Piperno G, Luck DJ. Paralyzed flagella mutants of Chlamydomonas reinhardtii. Defective for axonemal doublet microtubule arms. J Biol Chem. 1979;254(8):3091-9.

18. Omran H, Kobayashi D, Olbrich H, Tsukahara T, Loges NT, Hagiwara H, et al. Ktu/PF13 is required for cytoplasmic pre-assembly of axonemal dyneins. Nature. 2008;456(7222):611-16.

19. Mitchison HM, Schmidts M, Loges NT, Freshour J, Dritsoula A, Hirst RA, et al. Mutations in axonemal dynein assembly factor DNAAF3 cause primary ciliary dyskinesia. Nat Genet. 2012;44(4):381-9, s1-2.

20. Yamamoto R, Obbineni JM, Alford LM, Ide T, Owa M, Hwang J, et al. Chlamydomonas DYX1C1/PF23 is essential for axonemal assembly and proper morphology of inner dynein arms. PLoS Genet. 2017;13(9):e1006996.
